# Robust counterselection and advanced λRed recombineering enable markerless chromosomal integration of large heterologous constructs

**DOI:** 10.1093/nar/gkac649

**Published:** 2022-08-03

**Authors:** Dmitrii M Bubnov, Tigran V Yuzbashev, Andrey A Khozov, Olga E Melkina, Tatiana V Vybornaya, Guy-Bart Stan, Sergey P Sineoky

**Affiliations:** Bioresource Center Russian National Collection of Industrial Microorganisms (BRC VKPM), State Research Institute for Genetics and Selection of Industrial Microorganisms of National Research Center ‘Kurchatov Institute’ (NRC ‘Kurchatov Institute’ – GosNIIgenetika), 1-st Dorozhny pr., 1, Moscow 117545, Russia; Kurchatov Complex of Genetic Research, NRC ‘Kurchatov Institute’, Kurchatov Square, 1, Moscow 123098, Russia; Department of Bioengineering and Imperial College Centre for Synthetic Biology, Imperial College London, London SW7 2AZ, UK; Bioresource Center Russian National Collection of Industrial Microorganisms (BRC VKPM), State Research Institute for Genetics and Selection of Industrial Microorganisms of National Research Center ‘Kurchatov Institute’ (NRC ‘Kurchatov Institute’ – GosNIIgenetika), 1-st Dorozhny pr., 1, Moscow 117545, Russia; Kurchatov Complex of Genetic Research, NRC ‘Kurchatov Institute’, Kurchatov Square, 1, Moscow 123098, Russia; Department of Microbiology, Faculty of Biology, Lomonosov Moscow State University, Lenin's Hills 1-12, Moscow 119234, Russia; Kurchatov Complex of Genetic Research, NRC ‘Kurchatov Institute’, Kurchatov Square, 1, Moscow 123098, Russia; Laboratory of Bacterial Genetics, NRC ‘Kurchatov Institute’ – GosNIIgenetika, 1-st Dorozhny pr., 1, Moscow 117545, Russia; Bioresource Center Russian National Collection of Industrial Microorganisms (BRC VKPM), State Research Institute for Genetics and Selection of Industrial Microorganisms of National Research Center ‘Kurchatov Institute’ (NRC ‘Kurchatov Institute’ – GosNIIgenetika), 1-st Dorozhny pr., 1, Moscow 117545, Russia; Kurchatov Genomic Center, NRC ‘Kurchatov Institute’ - GosNIIgenetika, 1-st Dorozhny pr., 1, Moscow 117545, Russia; Department of Bioengineering and Imperial College Centre for Synthetic Biology, Imperial College London, London SW7 2AZ, UK; Bioresource Center Russian National Collection of Industrial Microorganisms (BRC VKPM), State Research Institute for Genetics and Selection of Industrial Microorganisms of National Research Center ‘Kurchatov Institute’ (NRC ‘Kurchatov Institute’ – GosNIIgenetika), 1-st Dorozhny pr., 1, Moscow 117545, Russia; Kurchatov Complex of Genetic Research, NRC ‘Kurchatov Institute’, Kurchatov Square, 1, Moscow 123098, Russia

## Abstract

Despite advances in bacterial genome engineering, delivery of large synthetic constructs remains challenging in practice. In this study, we propose a straightforward and robust approach for the markerless integration of DNA fragments encoding whole metabolic pathways into the genome. This approach relies on the replacement of a counterselection marker with cargo DNA cassettes via λRed recombineering. We employed a counterselection strategy involving a genetic circuit based on the CI repressor of λ phage. Our design ensures elimination of most spontaneous mutants, and thus provides a counterselection stringency close to the maximum possible. We improved the efficiency of integrating long PCR-generated cassettes by exploiting the Ocr antirestriction function of T7 phage, which completely prevents degradation of unmethylated DNA by restriction endonucleases in wild-type bacteria. The employment of highly restrictive counterselection and *ocr-*assisted λRed recombineering allowed markerless integration of operon-sized cassettes into arbitrary genomic loci of four enterobacterial species with an efficiency of 50–100%. In the case of *Escherichia coli*, our strategy ensures simple combination of markerless mutations in a single strain via P1 transduction. Overall, the proposed approach can serve as a general tool for synthetic biology and metabolic engineering in a range of bacterial hosts.

## INTRODUCTION

The progress across recent decades in developing methodologies for *Escherichia coli* genome engineering together with a comprehensive understanding of bacterial metabolism has enabled deep rewiring of cells by altering native systems and adding new functions. Expansion of cell capabilities relies on introducing potentially large heterologous metabolic pathways and genetic networks into the host cell genome. Thus, methods are required for the precise chromosomal integration of long heterologous DNA constructs. In the last twenty years, λRed recombineering has been demonstrated as a pivotal technology for *E. coli* genome engineering (1–5). This prominence is attributed to the ability of λRed recombination machinery to facilitate the recombination of linear DNA cassettes with homology arms as short as 50 bp, generated using one-step PCR. However, this system is commonly considered unable to promote the integration of fragments longer than 3–4 kb when using cassettes with a short flanking homology (6,7). This imposes severe constraints on using recombineering for delivering large DNA fragments to the chromosome. This obstacle can be partially solved by *in vitro* assembly of integrative cassettes that comprise extended homology arms, a cargo DNA, and a selectable marker on a plasmid vector (8,9). Constructing a plasmid with a positive selection marker and unique homology arms for each specific integration site significantly negates the flexibility and performance of λRed recombineering, and makes the whole procedure rather cumbersome.

The need for payload DNA to be joined with an antibiotic resistance marker can be circumvented by counterselection against non-recombinant cells instead of positive selection of recombinants. For example, I-SceI-mediated chromosome breakage combined with expression of λRed functions has been shown to provide markerless integration of heterologous DNA. In this case, a payload must still be delivered into the cell as part of a plasmid vector (7). Clustered regularly interspaced short palindromic repeats (CRISPR) technology (10,11) is closely related to the I-SceI-based strategy in its operation principle and dependence on λRed functions (12–15). CRISPR-based methods have been shown capable of promoting the insertion of linear PCR-generated constructs (12,14,16). However, the counterselection efficiency imposed by Cas9-mediated double-strand breaks can be highly variable depending on the sequence of gRNA used, the exact position of the target cleavage site relative to the origin of replication, and the promoter strength driving *cas9* transcription (17–19). The CRISPR approach has great potential for bacterial genome editing, especially in multiplexed engineering tasks. However, a complex set of experimental parameters must be carefully controlled to attain the delivery of large synthetic constructs to the chromosome with reasonable efficiency and robustness.

Although CRISPR-based technologies have been vigorously developed during the past decade, the conventional approach of genome engineering using λRed recombineering retains unexploited potential. Recombineering implies manipulation of the chromosome using a linear dual selectable cassette, which comprises an antibiotic resistance marker and coupled counterselectable marker (20–23). The counterselectable marker is typically considered an accessory genetic element that allows recycling of the antibiotic resistance gene and precise construction of markerless modifications. Simultaneously, an efficient counterselection strategy could enable replacement of an inserted cassette with any synthetic construct of interest by subsequent selection against cells that retain the counterselectable marker. Such an approach combines the marker elimination step with the delivery of desired constructs into the chromosome, thereby greatly accelerating the construction of strains with an extensively edited genome. The counterselection-based method could be more reliable and straightforward to use compared to CRISPR-based technologies. As this approach exploits the exclusive advantages of λRed recombineering as a well-established and robust method, there is no need for optimizing numerous experimental parameters and *in vitro* construction of a gRNA-expressing plasmid for each genomic locus to be targeted. Finally, using efficient counterselection provides a convenient method for the successive assembly of numerous mutations constructed in parallel through generalized P1 transduction (22,23). The only limitation of the proposed approach is the lack of counterselection markers to ensure a selection stringent enough to enable reliable discrimination of rare recombinants among numerous spontaneous mutants that escape killing.

To facilitate the introduction of large DNA fragments encoding heterologous metabolic pathways and genetic networks into the host cell genome, we aimed to develop a counterselection strategy by harnessing an artificial genetic circuit including the P_L_/P_R_ two-promoter system of bacteriophage λ and the CI repressor as a counterselectable marker. We used a circuit design to ensure efficient elimination of spontaneous escape mutants, and obtained stringent counterselection, which closely approached the maximum possible stringency level. When examining the insertion of PCR-generated constructs by replacing the counterselectable marker, the EcoKI restriction-modification system of the wild-type strain was found to impose a severe restraint on recombineering efficiency. However, co-expression of the *ocr* antirestriction function of the T7 phage with the λRed machinery to address this issue resulted in enhanced recombineering efficiency by over three orders of magnitude, thus facilitating providing the unobstructed delivery of unmethylated DNA into wild-type bacteria. Thus, we developed a streamlined and flexible genome engineering strategy relying solely on efficient counterselection and advanced recombineering, enabling the markerless integration of operon-sized cassettes generated by one-step PCR and flanked by short homology arms with an editing efficiency of 50–100% across arbitrary genomic sites. The proposed counterselection approach promotes P1 transduction of unmarked chromosomal loci between strains, thereby facilitating the simple assembly of numerous modifications in a single extensively engineered strain.

## MATERIALS AND METHODS

### Media and culture conditions

Bacteria were routinely grown in LB broth (10 g/l tryptone, 5 g/l yeast extract, 10 g/l sodium chloride) at 37°C, with shaking at 220 rpm. Solid LB medium was prepared by adding 20 g/l agar to LB broth. The medium was supplemented with ampicillin (200 mg/l), kanamycin (100 mg/l), gentamycin (20 mg/l), streptomycin (20 mg/l), or chloramphenicol (25 mg/l) as needed. The chloramphenicol concentration was increased to 200 mg/l in *cI-hok* counterselection experiments for strains harbouring the plasmid-borne P_L_-*cat* module. Counterselection against *rpsL* and *sacB* was performed using LB agar with 500 mg/l streptomycin or SuLB agar (10 g/l tryptone, 5 g/l yeast extract, 60 g/l sucrose). Carbohydrate utilisation was tested using LB agar supplemented with 25 mg/l 2,3,5-triphenyltetrazolium chloride and 1% of d-galactose, lactose, l-arabinose, or sucrose. Sucrose-utilizing recombinants were selected using M9 medium (24) with 0.5% sucrose.

### Molecular biology techniques and reagents

The oligos used for PCR and for recombineering were purchased from Evrogen Joint Stock Company (Moscow, Russia) as listed in Supplementary Table S1. Oligos longer than 80 nts were additionally purified using polyacrylamide gel electrophoresis by the manufacturer. Restriction endonucleases, T4 ligase, FastAp alkaline phosphatase, DreamTaq DNA polymerase, and T4 polymerase were purchased from Thermo Fisher Scientific Baltics (Vilnius, Lithuania). DNA fragments for cloning and recombineering were amplified using KAPA HiFi DNA Polymerase (Kapa Biosystems, Wilmington, USA).

### Bacterial strains and plasmids

All bacterial strains used in this study, including information on their genotype and phenotype, are presented in Supplementary Table S2. All *Escherichia coli* strains constructed throughout this study are derivatives of MG1655. Mutations specific to MG1655 (F^−^ λ^−^*ilvG*^−^*rfb*-50 *rph*-1) are omitted from the genotypes of its derivative strains. Strains were constructed by λRed recombineering (2,25) using oligonucleotides or integrative cassettes as recombination substrates. Mutations were transferred from strain to strain using P1 transduction. The exact methods for constructing each strain are summarised in Supplementary Table S2, including the parental strain, pair of primers and template used to amplify the integrative cassette, or, in the case of transduction, the pair of donor and recipient strains used, and information on the mutation to be transferred.

The plasmids used in this study, their complete sequence, and relevant characteristics are listed in Supplementary Table S3 and their detailed construction is described under ‘Plasmid construction’ in the Supplementary Materials and Methods.

### λRed recombineering

Plasmids pRedCm, pRedCmOcr or the chromosomally integrated *ocr-γβexo*-P_L_-*cat* module were employed for expressing the λRed recombination functions. The plasmid pDL17 (26) was used as a helper for integrating cassettes that confer chloramphenicol resistance. A detailed description for preparing electrocompetent cells and linear DNA cassettes is presented under ‘Preparation of linear cassettes’ and ‘Preparation of electrocompetent cells’ in the Supplementary Material and Methods. Electroporation was performed as follows. A 50 μl aliquot of electrocompetent cells induced for expressing λRed functions was mixed with 0.5–4 μl of the DNA sample and electroporated in 1 mm cuvettes using the Eppendorf Electroporator 2510 (Eppendorf AG, Hamburg, Germany) operated at 1.8 kV, 10 μF, and 600 ohm. Generally, 0.1–0.2 μg of *cat-sacB*, *rpsL-cat* or *cI-hok* cassettes were electroporated. Cells were then resuspended in 1 ml of LB and recovered for 2.5 h in a 15-ml polypropylene tube at 37°C with shaking at 220 rpm before plating onto a selective medium. If necessary, the medium was supplemented with 1 mM IPTG to inhibit helper plasmid replication. In case of *cI-hok* cassettes, the pRedCm or pRedCmOcr plasmids were retained and used as a reporter to verify the expression of functional CI by testing recombinant colonies for chloramphenicol sensitivity. For measuring the titre of viable cells, the recovered suspension was diluted 10^5^-fold and plated on non-selective LB agar. The recombinant locus structure was verified by colony PCR using DreamTaq DNA polymerase. Before testing, colonies were re-streaked to isolate single clones. Two pairs of primers were used to amplify regions spanning the junctions between genomic and heterologous sequences on both sides of an inserted fragment.

### *cI-hok* counterselection and identification of recombinants

The *ara::lux*, *trp* and *scr* cassettes were generated using PCR, whereas the *lac*::*vio* cassette was obtained by plasmid digestion as described under ‘Preparation of linear cassettes’ in the Supplementary Material and Methods. To render two EcoKI recognition sites within the *lac::vio* cassette susceptible to EcoKI digestion, the plasmid was extracted from EcoKI methyltransferase-deficient cells. The resulting linear fragment was dephosphorylated to make it identical to PCR-generated cassettes in our experiment. The complete sequence of all cassettes is presented in Supplementary Table S4.

Markerless cassettes were introduced by *cI-hok* cassette replacement and subsequent counterselection. Cells electroporated with 0.4–1 μg of the cassettes were recovered in 20 ml of antibiotic-free LB for at least 6 h (usually, overnight) to ensure CI exhaustion and *cat* derepression. Recombinants were selected on LB agar supplemented with chloramphenicol at either 200 mg/l for the multicopy P_L_-*cat* module within the helper plasmid or at 25 mg/l for the helper module in the chromosome. The medium was additionally supplemented with components required for direct detection and accounting of recombinant colonies that acquired the *scrKYABR*, *vioABCDE* or *luxCDABE* operon. Sucrose-utilizing recombinants were screened on chloramphenicol plates with 25 mg/l 2,3,5-triphenyltetrazolium chloride and 1% sucrose. Cells electroporated with the *luxCDABE* operon targeting the *ara* locus were first spread on chloramphenicol plates. Resistant colonies were randomly picked and transferred to a 96-well plate and replica plated on LB agar with 0.2% l-arabinose to induce expression. Luminescent recombinants were distinguished by imaging on the GelDoc Gel Documentation System (BioRad) with 5 min exposure in dark. Recombinants with the *vioABCDE* operon inserted downstream of the P*_lacZYA_* promoter were counted on selective plates with additional incubation at room temperature for 2–3 days until the recombinants turned deep violet because of violacein production. For integration with the B2141 strain, the medium was supplemented with 10 μM IPTG for recombinant selection. Recombinants that acquired the *trp* cassette were selected on chloramphenicol plates with 0.4% glucose to repress tryptophanase expression.

The protocols for both recombineering and *cI-hok* counterselection in *Salmonella*, *Citrobacter* and *Pantoea* were similar to those used in *Escherichia coli*, requiring only minor modifications, which are summarized in the Supplementary Table S8.

In all experiments, at least seven colonies exhibiting the recombinant phenotype were re-purified and tested using colony PCR for the desired mutation in a targeted genomic locus. The test was performed similar to that for verifying insertions of dual selectable cassettes.

## RESULTS

### Design of a counterselection strategy

A pivotal constituent element of our approach for delivering heterologous DNA into the *E. coli* genome is a counterselection strategy, which has to meet several requirements. The main trait is exceptional restriction of spontaneously arising mutants, which survive under selective conditions. We sought a counterselection marker that is easily portable, thereby enabling its use in any strain of interest without requiring extensive preliminary genome engineering. Additionally, the counterselection strategy must be as robust and straightforward as possible to minimise the need for strain-to-strain optimisation and to streamline the selection procedure. To satisfy these demands, similar to the previously described counterselection method used for the mutagenesis of *Bacillus subtilis* (27,28), we exploited the CI-repressible antibiotic resistance gene. We placed *cat* under the control of P_L_, whereby this transcription unit confers chloramphenicol resistance (Cm^R^) as long as the functional *cI* gene is absent. Otherwise, CI represses *cat* transcription and cells remain chloramphenicol-sensitive. Combined with the P_L_-*cat* module, *cI* serves as a counterselectable marker, which could be easily eliminated by selecting chloramphenicol-resistant cells. However, the P_L_-*cat* module first needs to be delivered into an unmodified host strain. Therefore, we cloned this transcription unit into a helper plasmid expressing λRed functions under control of the P*_rhaB_* promoter, thereby producing the pRedCm helper plasmid. Remarkably, this plasmid is based on the previously described pMB1-derived conditional origin of replication (26), whose activity is controlled by the LacI-repressible P_T5/_*_lacO_* promoter and is severely inhibited by IPTG supplementation. The pRedCm plasmid could be easily eliminated from host cells by two rounds of re-streaking as described under ‘Helper plasmid curing’ in the Supplementary Material and Methods.

**Figure 1.**
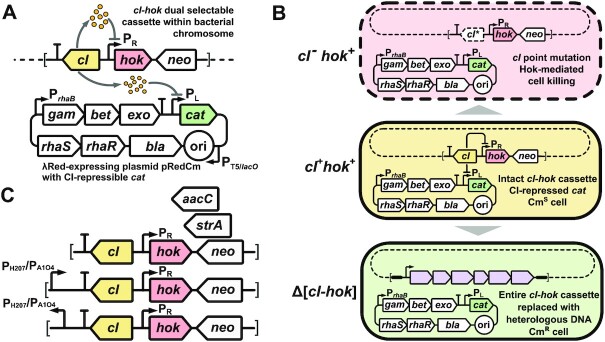
General outline of the *cI-hok* counterselection strategy and helper genetic constructs. (**A**) Illustration of the *cI-hok* counterselection principle. The *cI-hok-neo* cassette is delivered into the host genome by recombineering using the *neo* marker for positive selection and *rhaSR-*P*_rhaB_*-driven λRed functions provided by the pRedCm helper plasmid. Upon chromosomal integration, *cI* confers tight repression of P_R_-*hok* within the same dual selectable cassette and the P_L_-*cat* unit residing within pRedCm, by which the cell remains viable and chloramphenicol-sensitive. (**B**) Three possible fates of cells carrying the *cI-hok* cassette and the pRedCm plasmid. As long as functional *cI* is present, the cell remains viable and chloramphenicol-sensitive (middle scenario). Spontaneous mutation of *cI* alone results in derepression of both P_L_-*cat* and P_R_-*hok* units, subsequent Hok-mediated membrane permeabilisation, and death of the mutant (upper scenario). ‘En bloc’ elimination of the *cI-hok* cassette because of a purposive recombination event causes derepression of P_L_-*cat* and enables selection of chloramphenicol-resistant recombinants (bottom scenario). (**C**) A set of template constructs for amplifying *cI-hok* cassettes with *neo* (kanamycin), *strA* (streptomycin), or *aacC4* (gentamycin) orthogonal positive selection markers. The two bottom constructs carry either the P_H207_ (in truncated form) or P_A1O4_ promoter in front of the T_L3S1P56_ terminator. For each promoter, there are two alternative constructs in which the promoter is directed towards or away from the terminator.

The *cI* gene along with the plasmid-residing P_L_-*cat* module is an ordinary counterselection marker, which can be inactivated by numerous mutations. The second transcription unit is dedicated to eliminating these spontaneous mutants. It comprises the *hok* gene under control of the P_R_ promoter. This gene encodes a small membrane-depolarizing toxin, which is a part of the *hok-sok* postsegregational killing system of R1 plasmid (29,30). Therefore, the presence of P_R_-*hok* forces cells to express the functional CI repressor. We combined the P_R_-*hok* unit with *cI* and the kanamycin resistance gene as part of a single dual selectable *cI-hok-neo* cassette (Figure 1A). Once established, the resulting genetic circuit has three possible scenarios of further progression (Figure 1B): (i) stable maintenance of the functional *cI* and *hok* genes along with the repressed state of *cat*; (ii) spontaneous mutation of *cI* to inactivity causing simultaneous derepression of the P_L_-*cat* and P_R_-*hok* modules, with subsequent Hok-induced death of the mutant and (iii) ‘en bloc’ elimination of the *cI-hok* cassette because of a purposive recombination event such as the insertion of a heterologous DNA cassette, wherein *cat* derepresses and cells remain viable, upon which the recombinant can be selected for chloramphenicol resistance.

We anticipated that adjacent chromosomal promoters can cause transcriptional readthrough and unintended derepression of *hok*. Therefore, we placed a strong semisynthetic terminator T_L3S1P56_ (31) upstream of the *cI* gene C-terminus and verified that the terminator reliably protects the cassette from neighbouring transcription signals (see section 1 of Supplementary Results and Supplementary Figure S1), thereby obviating obstacles in inserting the *cI-hok* cassette into arbitrary genomic loci.

**Figure 2.**
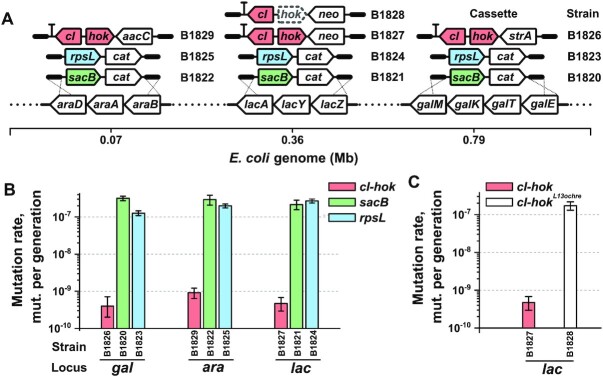
Evaluation of the efficiency of *cI-hok* counterselection. (**A**) Schematic representation of the construction of strains involved in the measurement of mutation rates to counterselection resistance. Each of the *galETKM, lacZYA* and *araBAD* operons was targeted in MG1655 with either the *cI-hok* or *cat-sacB* cassettes using the same homology arms for each locus. The *cat-rpsL* cassettes were inserted into the chromosome of B1733 (MG1655 *rpsL^K43R^*). The *lacZYA* operon was additionally targeted using the *cI-hok^L13ochre^-neo* cassette with inactive *hok*. (**B**) Mutation rate to counterselection resistance for *cI-hok, sacB* and *rpsL* markers. The *cI-hok* strains were transformed with pRedCm to enable counterselection. Spontaneous mutants were selected on LB with chloramphenicol (200 mg/l), LB with streptomycin (500 mg/l), and SuLB plates for *cI-hok*, *rpsL* and *sacB* strains, respectively. The values shown and expressed as mutations to counterselection resistance per generation were calculated from fluctuation assay data (the frequencies of the mutants are shown in Supplementary Figure S4) using the Ma-Sandri-Sarkar maximum likelihood method implemented in the FluCalc web tool as described in the section ‘Measurement of mutation rate to counterselection resistance’ of the Supplementary Materials and Methods. Error bars indicate 95% confidence intervals calculated using the same software. In experiments with B1826 [pRedCm], a portion of the cultures did not contain Cm^R^ mutants. Thus, the mutation rate was calculated using the *p*0 method and 95% confidence intervals were determined as described in (36). (**C**) Comparison of mutation rates of the *cI-hok* and *cI-hok^L13ochre^* within the *lac* locus. All measurements and calculations were performed as described for Figure 2B.

The scope of the *cI-hok* counterselection strategy was expanded by providing additional cassettes (Figure 2C). We replaced the kanamycin resistance gene with two orthogonal markers conferring resistance to gentamycin (*aacC4*) and streptomycin (*strA*). To make *cI-hok* constructs relevant for metabolic engineering we constructed four cassettes containing the strong P_H207_ or P_A1O4_ promoters (32,33) adjacent to the T_L3S1P56_ terminator (Figure 1C). In a particular construct, each of these promoters is either directed away from or towards the cassette. The first type of cassette is intended to be inserted in front of chromosomal genes thereby replacing their native promoters. The second one, once is inserted into a desired genomic locus, can be substituted with a promoterless construct of interest via *cI-hok* counterselection. By this manipulation, the construct is placed under the control of the P_H2O7_ or P_A1O4_ (for more detail see Supplementary Figure S2).

### Evaluation of the efficiency of *cI-hok* counterselection

To accurately estimate the efficiency of the *cI-hok* approach, we determined the most stringent selection conditions, i.e. the maximal concentration of chloramphenicol in LB agar that MG1655 with pRedCm (in which the P_L_-*cat* module is completely derepressed) could tolerate without reduced plating efficiency. The threshold concentration was found to be 200 mg/l and was used for further experiments (Supplementary Figure S3).

As shown by Luria and Delbruck, owing to the stochastic nature of mutation events in a bacterial population, the frequency of mutants may vary by orders of magnitude in each independent culture when the inoculum is initially small enough and does not contain mutants (34). Therefore, the frequency of mutant occurrence does not adequately reflect the robustness of counterselection approaches. Therefore, we used a mutation rate to counterselection resistance as an estimate of efficiency and compared the *cI-hok* strategy with the two most widely used approaches, which utilise *sacB* (5,35) and *rpsL* (20) as markers. For this, we chose three operons, *galETKM*, *lacZYA* and *araBAD*. Each of them was replaced with the *cI-hok, cat-sacB* or *cat-rpsL* dual selectable cassette in parallel using the same flanking homology regions (Figure 2A). Using the resulting strains, we performed a fluctuation assay to estimate mutation rates to counterselection resistance for these three markers (see Supplementary Figure S4 for frequencies of occurrence of mutants). The *cI-hok* cassette in conjunction with the P_L_-*cat* module on pRedCm exhibited a mutation rate of 4 × 10^−10^–9 × 10^−10^ mutations per generation (Figure 2B) depending on the genomic locus of cassette insertion. Comparison of mutation rates with that of *sacB* and *rpsL* markers inserted into the same loci revealed that the values of *cI-hok* were 320–790 times lower compared with the corresponding values of *sacB*, and 220–570 times lower compared with those of *rpsL*.

We verified that the P_R_-hok transcription unit is the essential element responsible for the high efficiency of the *cI-hok* strategy. We constructed the *cI-hok*^L13ochre^*-neo* cassette where *hok* was inactivated by the premature ochre codon. We inserted this cassette into the *lac* locus and found that disruption of the *hok* coding sequence leads to a 360-fold increase in mutation rate to chloramphenicol resistance compared to that for the cassette with the wild-type *hok* (Figure 2C). This result indicates that, as expected, the P_R_-*hok* module efficiently eliminates most of the spontaneous *cI* mutants.

### Restriction alleviation facilitates integration of long PCR-amplified constructs

To probe the applicability of *cI-hok* counterselection for delivering heterologous constructs into the genome, we sought to perform direct λRed-mediated markerless integration of long PCR-amplified DNA fragments by replacing the *cI-hok* cassette and selecting recombinants for chloramphenicol resistance. To ensure simple measurement of replacement efficiency, we chose the *scrKYABR* and *luxCDABE* operons that confer easily observable phenotypes upon their expression (Supplementary Figure S5A and B). We amplified both fragments with short flanking homology regions of 80 bp targeting the desired genomic loci. The *scrKYABR* and *luxCDABE* fragments targeted the *gal* and *ara* loci, respectively. In preliminary experiments with the *gal::scrKYABR* cassette, we could isolate recombinant colonies via *cI-hok* counterselection. However, the rate of recombinants among spontaneous Cm^R^ mutants was only 1–3%, whereas replacing the *cI-hok* cassette in the *ara* locus with the *luxCDABE* operon consistently yielded >60% of positive recombinants in several independent experiments. This was especially surprising as the efficiency of the *cI-hok* counterselection in the *ara* locus was the lowest among the three loci tested (Figure 2B). Sequence inspection of the cassettes showed that *scrKYABR* comprises three recognition sites for EcoKI endonuclease, whereas *luxCDABE* has none. Although previous studies have shown that EcoKI does not affect λRed recombineering with a PCR-generated cassette (5), we hypothesised that the disparity between the *gal::scrKYABR* and *ara::luxCDABE* cassettes likely reflects EcoKI-mediated fragmentation of foreign unmethylated DNA. Considering the relatively high abundance of EcoKI recognition sites (1 per 8 kb DNA sequence), this endonuclease can drastically hinder genome manipulation using long PCR-generated substrates. To overcome this barrier, we explored the capability of several phages and conjugative plasmids to alleviate the restriction imposed by the host. To find the best candidate, we probed several antirestriction functions from different sources, i.e. Ral of λ phage, ArdA of the plasmid ColIb-P9, and Ocr of T7 phage. The last two proteins inhibit the EcoKI endonuclease due to DNA mimicry (37,38), whereas Ral acts by stimulating EcoKI methyltransferase activity on unmodified DNA (39).

**Figure 3.**
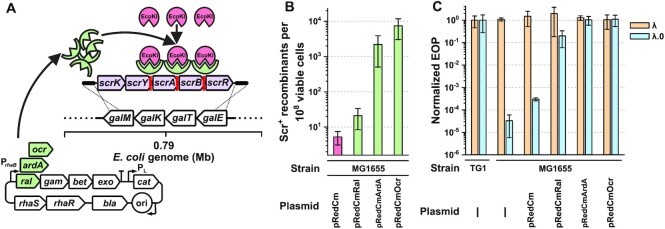
Stimulation of λRed recombineering by suppression of EcoKI endonuclease activity. (**A**) Schematic of the experiment for assessing the capability of antirestriction functions to improve recombineering efficiency. The *ral, ardA*, or *ocr* genes were cloned on pRedCm upstream of *gam*. The resulting plasmids were transformed into MG1655 and tested as helpers for replacement of the *galETKM* operon with the *scrKYABR* cassette, which includes three EcoKI recognition sites (shown as red rectangles). (**B**) Efficiency of replacing the *galETKM* operon with the *scrKYABR* cassette. MG1655 cells carrying pRedCm, pRedCmRal, pRedCmArdA, or pRedCmOcr were induced for expression of λRed and antirestriction functions and electroporated with 0.6 μg of the *scrKYABR* cassette. Scr^+^ recombinants were selected on M9 plus 0.5% sucrose plates and the viable cell titre was determined on non-selective LB plates. The values shown are the average of three biological replicates; error bars indicate SD. (**C**) Effect of antirestriction functions on the efficiency of plating (EOP) of λ and unmethylated λ.0 phages. A detailed description is presented under ‘Assay of λ phage plating efficiency’ in the Supplementary Materials and Methods. Both phages were plated on cells induced for expressing λRed and antirestriction functions as well as on cells of plasmidless MG1655 and TG1 strains. Normalised EOP for each strain was calculated by dividing the determined phage titre by the titre of the same phage on a lawn of TG1. The average and SD were then calculated using the normalised EOP. For TG1, normalised SD was calculated by dividing non-normalised SD by the non-normalised average. The values shown are the averages of three biological replicates; error bars indicate SD.

We introduced these antirestriction genes into pRedCm upstream of *gam*, so that *ral, ardA, ocr* and Red genes constitute a single artificial operon under the control of P*_rhaB_*, thereby generating pRedCmRal, pRedCmArdA, and pRedCmOcr (Figure 3A). We transformed these plasmids as well as pRedCm into MG1655 and measured the efficiency of λRed-mediated recombineering using the *gal::scrKYABR* cassette, which we assumed to be highly susceptible to EcoKI action. We found that electroporation of cells expressing the λRed genes alone yielded only a few recombinants (Figure 3B). Co-expression of Ral had little to no effect on the efficiency of recombineering. Unlike Ral, ArdA and, to a higher extent, Ocr, provided a drastic increase in the yield of Scr^+^ recombinants. Specifically, co-expression of Ocr with Red proteins stimulated the recombination rate by over three orders of magnitude. We then explored whether suppression of EcoKI endonuclease activity was complete, i.e. whether the highest possible recombineering efficiency had been achieved. To do this, we assayed the efficiency of plating (EOP) of unmethylated λ.0 phage (Figure 3C). In this experiment, the non-restricting TG1 and wild-type MG1655 strains served as the positive and negative control, respectively. The upward tendency of the EOP of λ.0 on host cells expressing Ral, ArdA or Ocr was found to resemble that of the recombineering efficiency, whereas the EOP of modified λ remained unaffected. These results indicate that the activity of EcoKI endonuclease was the only obstacle for delivering *gal::scrKYABR* into the chromosome. Considering that co-expression of Ocr together with Red completely abolishes the restriction of λ.0 imposed by EcoKI, we concluded that the recombineering efficiency plateaued in *ocr-*expressing cells.

### Rational improvement of *cI-hok* counterselection by spontaneous escape mutant analysis

Having enhanced the integration efficiency of long PCR-generated cassettes, we examined whether it is possible to further improve the efficiency of *cI-hok* counterselection. Although the *cI-hok/*P_L_*-cat* genetic circuit was designed to be refractory against inactivating mutations, we could still isolate spontaneous mutants, which fall into two predominant classes (for more details see section 2 of Supplementary Results and Supplementary Figure S6). The first class of these mutants corresponds to spontaneous deletions encompassing the *cI-hok* cassette. Furthermore, some deletions extend to the surrounding chromosomal regions at least 6–9 kb apart from the cassette. This class accounted for 28–46% of the total number of mutants depending on the site of *cI-hok* integration (*gal*, *lac*, or *ara*). Most of the remaining mutations involved the pRedCm helper itself. These mutations correspond to tandem duplication of *cat* so that the second copy was placed directly downstream of the P_T5/lacO_ promoter. It appears that *cat* can be expressed through the basal activity of P_T5/lacO_ in these mutants. The last type of mutants indicated some potential for further reducing the mutation rate. Remarkably, the mutant plasmids resemble the product of an inappropriately recirculated linear multimer known to be produced upon expression of λRed genes (40,41). This suggests that the high copy number of pRedCm is a major drawback. First, pRedCm appears to cause some low-level expression of λRed, which occasionally induces formation of such multimers and concomitant emergence of defective plasmids in which *cat* is no longer repressed by CI. Second, the presence of many plasmid copies in the cell provides a multiplicity of target sites that could be subjected to mutation. To reduce both the copy number and uninduced expression level of λRed, we transferred the entire *ocr-γβexo-*P_L_-*cat* helper module into the chromosome. Further, to achieve strong repression and convenient induction of the *ocr-γβexo* operon, we engineered a promoter/repressor cassette comprising a strong hybrid P_A1lacO-1_ promoter (42) and *lacI* overexpressed under the control of P_H207_. We verified strong repression using the *lux* reporter (Supplementary Figure S7) and integrated the *ocr-γβexo-*P_L_-*cat* module under the control of P_A1lacO-1_, replacing the *araC-araBAD* chromosomal region. A threshold concentration of chloramphenicol for the resulting B2137 strain was determined as for the pRedCm helper and was found to be 25 mg/l.

Finally, we combined the chromosomal helper module with the Δ*galETKM::cI-hok-strA* and Δ*lacZYA::cI-hok-neo* mutations generating the B2140 and B2141 strains, respectively, and measured the mutation rates to counterselection resistance. In experiments with the B2140 strain, we could not select a single Cm^R^ mutant among 20 independent cultures containing 3.4 × 10^9^ cells each. For B2141, the mutation rate was determined as 9.9 × 10^−11^ mutations per generation (95% confidence interval 3.5 × 10^−11^–2.2 × 10^−10^), which is approximately 4-fold lower than that of the isogenic strain, B1827, with the pRedCm helper. We isolated 16 independent mutants and analysed them as described in section 2 of Supplementary Results. Among these, 15 lost kanamycin resistance and 13 could not utilise propionate, indicating disruption of the *prpBCDE* operon, which is 6 kb apart from the cassette. We thus inferred that, when using the chromosomal helper construct, counterselection escape mutants emerge almost exclusively because of intrachromosomal deletions of the *cI-hok* cassette. Low-frequency intrachromosomal deletions are an intrinsic feature of the *E. coli* genome, and their emergence is unlikely to be readily manipulated without modification of the replication and recombination machinery (43,44). As most mutants originate from spontaneous deletions of the *cI-hok* cassette we concluded that the highest possible efficiency of counterselection was achieved.

### Chromosomal integration of large constructs and transfer of markerless genomic loci through *cI-hok* counterselection

**Figure 4.**
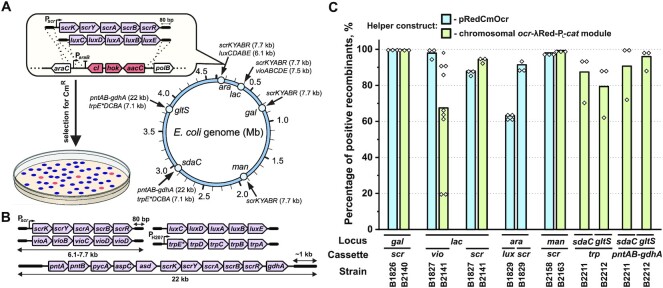
Markerless integration of linear constructs via *cI-hok* counterselection. (**A**) Schematic of experiments for estimating the efficiency of markerless cloning. Upon electroporation, a linear cassette (shown in violet) replaces the *cI-hok* cassette within a specific locus (*ara* in the figure) via λRed recombination, thereby enabling selection of recombinant colonies for Cm^R^ due to derepression of the P_L_-*cat* module within pRedCmOcr or the chromosomal *ocr-γβexo-*P_L_*-cat* helper construct. Targeted genomic loci are indicated as open circles along the chromosome. The value in parentheses near the name of a cassette indicates its entire length. (**B**) Schematic of the construct used for estimating the efficiency of markerless integration. The total length of the cassettes and flanking homology arm are indicated. The cassettes were prepared as described under ‘Preparation of linear cassettes’ in the Supplementary Materials and Methods (also see Supplementary Figure S14). An asterisk in the designation ‘trpE*’ refers to the S40F amino acid substitution in the anthranilate synthase, which renders the enzyme insensitive to feedback inhibition by l-tryptophan. (**C**) The efficiency of markerless cassette integration across genomic loci. Briefly, induced cells were electroporated with 0.4–1 μg of a cassette, recovered overnight in LB and plated on LB plus chloramphenicol plates with supplements enabling discrimination of recombinants as described under ‘*cI-hok* counterselection and identification of recombinants’ in the Materials and Methods. Colonies exhibiting the desired phenotype (sucrose utilisation, violacein production, or luminescence) were recognised as recombinants. For each cassette, at least seven recombinant colonies were purified and used for verifying the construct presence within a targeted locus by a locus-specific PCR (Supplementary Figure S8). In experiments with the *trp* cassettes, 16 Cm^R^ colonies per biological replicate were tested using locus-specific PCR with no phenotypic screening. The presence of the *pntAB-gdhA* module in the *sdaC* and *gltS* loci was additionally verified by Sanger sequencing of the junctions between the construct and chromosome. The percentage of positive recombinants was calculated by dividing the titre of recombinants by the total number of Cm^R^ colonies. The exact numerator and denominator are listed in Supplementary Table S5. The bars indicate average efficiency of integration, and open diamonds indicate exact values for each biological replicate.

We probed the performance of the established counterselection strategy and λRed recombineering for markerless delivery of high-capacity constructs into the genome. Specifically, we examined the replacement efficiency of *cI-hok* cassettes inserted into four distinct chromosomal loci, i.e. *gal, lac, ara* and *man* (*ΔmanXYZ*::*cI-hok-neo*) (Figure 4A). Replacement experiments were performed using the *scrKYABR, luxCDABE*, or *vioABCDE* cassettes flanked by homology regions of 72–82 bp length.

Using the pRedCmOcr helper and the chromosomal *ocr-γβexo-*P_L_*-cat* module, we observed a generally high integration efficiency of 60–100% (Figure 4C). These results indicate that the frequency of spontaneous mutants escaping counterselection was low compared to that of desired recombinants. Remarkably, PCR analysis revealed that even a fraction of clones recognised as non-recombinants according to phenotypic screening successfully recombined the cassettes into the targeted loci. Specifically, 9 of 24 non-luminescent colonies and 10 of 10 Scr^−^ colonies isolated in the experiments with the *ara::luxCDABE* and *gal::scrKYABR* cassette had the construct inserted into the desired locus (Supplementary Figure S8). Expression of the proper phenotype in these clones seems to be impaired by a mutation introduced through PCR amplification. Notably, each replicate started from different colonies. Therefore, the variability in the occurrence of spontaneous mutants among independent cultures was considered.

One can assume that the estimated efficiency of integration might be distorted because of the long outgrowth of electroporated cells in the liquid medium required for CI exhaustion and *cat* derepression. Indeed, a faster growth of desired recombinants compared with that of a parental strain could result in overestimation of the apparent percentage of positive colonies. To exclude this possibility, we measured the growth rates for each pair of parental and derivative strains with the *scr, vio*, and *lux* cassette inserted in the chromosome; however, we did not find pairs for which the ratio of growth rates favored overestimation of the percentage of recombinants (Supplementary Figure S9).

Next, we examined whether unmarked genomic loci can be transferred between strains via *cI-hok* counterselection using P1 transduction or electroporation with the fragmented genomic DNA of a donor strain also referred to as generalised allelic exchange (23). Using these approaches, we attempted to restore the *galETKM, lacZYA, araBAD*, or *manXYZ* operons in strains where these loci were disrupted by *cI-hok* cassette insertion. We observed that the percentage of positive recombinants reached 80–100% among Cm^R^ colonies (Supplementary Figure S10; Tables S6 and S7) with a few minor exceptions (see section 3 of Supplementary Results and Supplementary Figure S11).

### *cI-hok* counterselection is a useful tool for metabolic engineering

We demonstrated the applicability of *cI-hok* counterselection for creating strains that produce two industrially manufactured amino acids, L-tryptophan and L-threonine. For constructing an L-tryptophan producer, we integrated the P_H207_-*trpE*^S40F^*DCBA* operon into the *gltS* and *sdaC* loci in parallel (Figure 4A). This construct encodes the structural genes for the L-tryptophan biosynthetic pathway (Supplementary Figure S12A) with several additional mutations relative to the wild-type sequence. Specifically, we replaced the native promoter and *trpL* attenuator with the strong constitutive P_H207_ promoter and introduced the S40F amino acid substitution to the coding sequence of *trpE*, which render the anthranilate synthase insensitive to feedback inhibition by L-tryptophan (45). Upon electroporation of the recipient B2211 and B2212 strains we found that 63–94% of Cm^R^ colonies had the *cI-hok-neo* cassette in the targeted locus replaced with the P_H207_-*trpE*^S40F^*DCBA* construct (Figure 4C). A test-tube fermentation revealed that the resulting recombinants accumulated 1.4–1.5 g/l of l-tryptophan (Supplementary Figure S12B).

To improve the biosynthetic capacity of the l-threonine producing strain, we constructed a *pntAB-gdhA* cassette of 22 kb length (Figure 4B), containing six genes (*pntAB*, *gdhA*, *aspC*, *asd* and *pycA*) that control the biosynthetic reactions of the L-threonine biosynthesis pathway as well as the NH_4_^+^ assimilation and NADPH regeneration reactions (Supplementary Figure S13A). Additionally, this module contains the *scrKYABR* operon that enables sucrose utilization and serves as a phenotypic marker. We prepared the linear cassette with homology arms of ∼1 kb in length by plasmid digestion, and used this cassette to replace the *cI-hok-neo* cassette in either the *sdaC* or *gltS* gene by applying the chromosomal helper. We observed ∼50–200 Cm^R^ colonies compared to thousands in case of the shorter cassettes, indicating that *pntAB-gdhA* closely approaches the upper limit for the length of linear cassettes that can be reliably integrated through λRed recombineering. Nonetheless, 72–100% of Cm^R^ colonies were correct recombinants regardless of the targeted locus (Figure 4C). Next, we transferred the *ΔsdaC::cI-hok-neo* mutation to the L-threonine producing strain B2122 with subsequent replacement with the *ΔsdaC*::*pntAB-gdhA* construct via two-step P1 transduction. Upon fermentation, we found more than 50% improvement in L-threonine accumulation with the resulting strain (Supplementary Figure S13B).

### Adapting *cI-hok* counterselection and *ocr*-assisted recombineering to non-conventional enterobacteria

**Figure 5.**
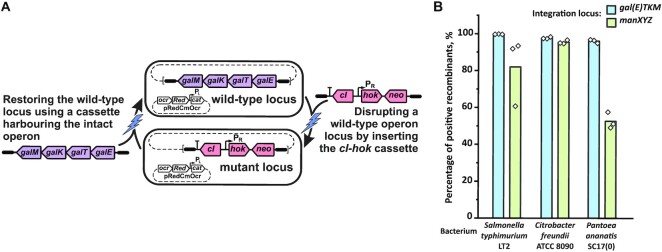
*cI-hok* counterselection and *ocr-*assisted recombineering in non-conventional bacteria. (**A**) Schematic of experiments for estimating the efficiency of markerless cloning. Either the *galETKM* (*galTKM* in the case of *Pantoea*) or *manXYZ* operon was replaced with the *cI-hok-neo* cassette. Next, the *cI-hok-neo* cassette was replaced with a PCR-amplified construct carrying the wild-type operon. (**B**) The efficiency of markerless cassette integration in the *gal* and *man* loci of the *Salmonella*, *Citrobacter*, and *Pantoea* genomes. *cI-hok* counterselection and λRed recombineering were performed using pRedCmOcr, pRedCmOcr^SC101ts^ and pRedCmOcr^RSF^ helper plasmids for *Citrobacter*, *Salmonella*, and *Pantoea*, respectively. Both these techniques were implemented similarly to the experiments in *E. coli* with minor modifications, which are described in the section ‘Recombineering and *cI-hok* counterselection in *Salmonella*, *Citrobacter*, and *Pantoea*’ of the Supplementary Material and Methods. The recombinants were selected for Cm^R^ on tetrazolium agar supplemented with either 1% d-galactose or 1% mannose. Colonies exhibiting either the Gal^+^, or Man^+^ phenotype were recognised as recombinants. For each cassette, seven recombinant colonies were purified and used for verifying the construct presence within the targeted locus using locus-specific PCR (Supplementary Figure S8). The percentage of positive recombinants was calculated by dividing the titre of recombinants by the total number of Cm^R^ colonies. The exact numerator and denominator are listed in Supplementary Table S5. The bars indicate average efficiency of integration, and open diamonds indicate the exact values for each biological replicate.

Finally, we explored whether the proposed genome engineering strategy can be employed to engineer bacteria other than *E. coli*. We examined its applicability to three distinct organisms, *Salmonella typhimurium* LT2 (46), *Citrobacter freundii* ATCC 8090 (47) and *Pantoea ananatis* SC17(0) (48,49). To accomplish this, we only had to make minor changes to our methods. Specifically, we found that the pRedCmOcr helper was unable to replicate in *Salmonella* and *Pantoea* cells, whereas *Citrobacter* cells were successfully transformed with this plasmid. To enable counterselection and recombineering in *Salmonella* we subcloned the entire *ocr*-*γβexo*-P_L_-cat module under control of the P_A1lacO-1_ promoter and LacI repressor from the chromosome of the B2137 strain to the pSC101^ts^ origin of replication. For manipulating the *Pantoea* genome, we subcloned the same construct, but the plasmid backbone was replaced with the wide-host-range RSF1010 origin and streptomycin-resistance marker *strA*. Using these helper constructs, we successfully deleted the *galETKM* (*galTKM* in the case of *Pantoea*) and *manXYZ* operons in all three bacteria by inserting the *cI-hok-neo* cassette. Subsequently, we restored the deleted operons by introducing corresponding constructs via electroporation of the mutant cells with PCR-amplified cassettes 3.5–4.5 kb in length, containing flanking homologous sequences of 80 bp. The percentage of the positive recombinants was found to be 50–100% (Figure 5).

## DISCUSSION

Since the year 1998, establishment of recombineering (50,51) has revolutionised genome engineering techniques by providing a flexible and straightforward way for constructing chromosomal mutations with single base-pair precision through integration of dual selectable cassettes, followed by their ‘scarless’ removal using counterselection markers. This last step is generally considered burdensome; however, it facilitates the markerless integration of desired constructs via selection against non-recombinant cells. On the condition that markerless cassettes can be generated by one-step PCR and inserted using short homology arms, the superposed elimination of a marker and chromosomal integration of heterologous constructs constitute a powerful approach for bacterial genome engineering relying solely on the advantages λRed recombineering as a well-established and versatile technology.

An ideal counterselectable marker needs to be exceedingly refractory against inactivating mutations to enable facile discrimination of recombinants among spontaneous mutants. For this, we exploited the ability of CI repressor from λ phage to serve as a counterselection marker when used in conjunction with the P_L_-*cat* module. Combining *cI* with the P_R_-*hok* transcription unit within a single cassette ensures efficient elimination of most deleterious mutations of *cI*, thereby reducing the mutation rate towards counterselection resistance by more than two orders of magnitude compared to that of *cI* alone. Similarly, comparison with commonly used markers, *sacB* and *rpsL*, showed that *cI-hok* counterselection is almost three orders of magnitude more restrictive. Besides these markers, several counterselection approaches have been described in recent years. Among these, the strategy involving toxins as counterselectable markers ensures high selection stringency and, like *cI-hok*, does not require extensive strain modification. Two distinct implementations of this approach have been described. The first involves the use of constitutively expressed *ccdB* toxin (52). The viability of *ccdB-*carrying cells is maintained owing to the presence of the cognate antitoxin, *ccdA*, under the control of an inducible promoter. Once this promoter is repressed, the cell dies because of *ccdB* expression as it is no longer protected by CcdA. The second strategy utilizes variable toxic genes directly under the control of an inducible promoter (22). The cells remain viable as long as the promoter is not induced but die once inoculated into an inducer-supplemented media. As these two strategies are highly similar, we consider that their efficiencies are nearly equal. The efficiency of the second implementation approach has been carefully evaluated. Specifically, the lowest mutation rate to counterselection resistance was found to be 1.42 × 10^−8^ mutations per generation which is 15-fold higher than our highest mutation rate (9.0 × 10^−10^), characteristic of the B1829 strain combined with the pRedCm helper. Inducible toxins are the most common class of counterselectable markers, which being a single conditionally lethal gene, could be easily inactivated by numerous point mutations. This constrains the maximum possible efficiency, which depends on the rate of genome replication errors and length of the marker, i.e. the number of available mutation sites. According to theoretical estimations performed by the authors who developed the inducible toxins-based approach for their most effective negative selection cassette of 811 bp length, a mutation rate to spontaneous selection resistance below 9.0 × 10^−9^ is unlikely. Several attempts to overcome the limitation of single-marker negative selection strategies have been described in literature. The *tolC* gene has been used as a marker for negative selection (53), conferring sensitivity to colicin E1 and vancomycin, but requires extensive preliminary strain engineering to enable high-efficiency counterselection. First, native *tolC* has to be inactivated. To achieve the highest selection stringency the authors duplicated the *tolQRA* region. Using the resulting strain, the authors reported the lowest observed frequency of mutants, resistant to both colicin E1 and vancomycin, to be 4.3 × 10^−11^. Duplication of a counterselection marker has also been implemented for developing a strategy utilizing the thymidine kinase gene (*hsvTK*) of the herpes simplex virus (54). Selection against *hsvTK*-harbouring cells is imposed because HsvTK induces lethal mutagenesis in presence of the synthetic nucleoside dP. Duplication of *hsvTK* allowed the authors to achieve a frequency of counterselection survivors of <1.67 × 10^−10^. However, dP induces 10-fold higher mutagenesis even in cells that have lost *hsvTK*, rendering such an approach impractical for precise genome engineering. For these two approaches, only the frequency of spontaneous mutants has been reported and the high variability of this value across independent cultures caused by the stochastic emergence of mutations has not been considered. Therefore, it is difficult to precisely compare our proposed *cI-hok* counterselection with their results. Nonetheless, using the B2140 strain harbouring the P_L_-*cat* module within its chromosome, we did not observe spontaneous mutants among 6.8 × 10^10^ cells (20 cultures, each containing 3.4 × 10^9^ cells). This gives us a mutant frequency value of less than 1.47 × 10^−11^, which is 3- and 11-fold lower than that reported for *tolC* and *hsvTK*, respectively. To the best of our knowledge, these markers are the most efficient among those reported to date. Through this comparison, we concluded that the *cI-hok* counterselection is the most stringent and robust. Indeed, when using the chromosomally integrated *ocr-γβexo-*P_L_-*cat* helper module, spontaneous mutants that survived the *cI-hok* counterselection arise almost exclusively due to intrachromosomal deletions which encompass the *cI-hok* cassette and adjacent genomic regions. These deletions are known to occur on both *recA^+^* and *recA^−^* genetic backgrounds and may not involve explicit sequence determinants such as direct repeats (43,55). Therefore, the rate of deletions may not be easily manageable. Considering this reasoning we concluded that the *cI-hok* strategy reaches almost the maximum possible efficiency for counterselection systems regardless of their type. Furthermore, the *cI-hok* counterselection design provides substantial advantages over existing counterselection systems in terms of handling. Specifically, selection for chloramphenicol resistance is a common and robust procedure that does not require expensive or commercially unavailable chemicals. Our approach only requires minimal strain-to-strain optimisation for determining an adequate threshold antibiotic concentration.

Recently, CRISPR/Cas9-based methods have been sought to develop markerless integration into the genome. To achieve reasonable efficiency, most approaches require providing a donor DNA within a plasmid vector (56), elongating homology arms to 400–600 bp (14,57–59), or fusing the fragment of interest with a positive selection marker (13,60). The necessity for high-efficiency homologous recombination was recently alleviated through establishment of the INTEGRATE system, which utilizes CRISPR RNA-guided transposons (61). This technology ensures integration of high-capacity cargo across distinct genomic sites but suffers from re-mobilisation of pre-existing inserts, which also confer target immunity, thereby hindering iterative integrations close to each other. Among homologous recombination-based methods, which provide seamless and precise integration as opposed to transposon-based technologies, the most powerful Cas9-based approach has been described by Basalo *et al.* (16). Using this method, a 10 kb PCR fragment with homology arms of 100 bp in length can be integrated into the genome. However, successful integration of such a long cassette has been shown exclusively at a single specific site (called Safe Site) where efficient Cas9-mediated cleavage is known to occur. Remarkably, the BW25113 strain used in this study is deficient for EcoKI endonuclease owing to the *hsdR514* mutation (62). It is unclear how this strategy works in wild-type strains wherein the restriction barrier inevitably becomes a bottleneck for cloning fragments longer than 8 kb. Our proposed approach solves this problem, thereby enabling facile cloning of long PCR-amplified heterologous constructs in wild-type bacteria. Co-expression of the *ocr* function from T7 phage and λRed recombination machinery combined with extremely stringent *cI-hok* counterselection allowed markerless integration of operon-sized cassettes with short homology arms across arbitrary genomic loci. Extending the homology arms of the integration cassette allowed us to insert a cluster of genes, 22 kb in length, with an efficiency of almost 100%. Taken together, these results indicate that the performance of our proposed genome engineering strategy, which utilizes *cI-hok* counterselection, is limited by the efficiency of λRed-recombineering and electroporation rather than by the reliability of counterselection itself. Furthermore, the *cI-hok* strategy seems more streamlined compared to CRISPR-based methods, which usually utilise two (14,56,63) or three (12,13,16) distinct plasmids to support the expression of Cas9, gRNA, and λRed. In contrast, the *cI-hok* counterselection method only requires a single conditionally replicating helper plasmid, which can be easily eliminated from the strain. Additionally, when using the pRedCmOcr helper, our strategy does not require preliminary strain modification to enable counterselection and can be implemented for virtually all wild-type or engineered strains. The only prerequisite for a strain is to be free from the λ prophage and the chloramphenicol resistance marker. Alternatively, to achieve the maximum counterselection efficiency, a strain of interest can be equipped with the chromosomally integrated *ocr-γβexo*-P_L_-*cat* helper module and used with no plasmids at all. This construct can be easily introduced via P1 transduction and can be eliminated by transducing the wild-type locus and selecting for L-arabinose utilisation.

Upon implementation of the *cI-hok* strategy for the integration of heterologous constructs, each targeted genomic locus has a counterpart marked by an antibiotic resistance gene. This provides an opportunity to assemble multiple mutations in a single extensively engineered strain. Indeed, we demonstrated the possibility of transferring a markerless locus via P1 transduction and selection of recombinants in which this locus replaced the *cI-hok* cassette. Alternatively, the same result could be accomplished with even higher efficiency by the transformation of a recipient strain with the fragmented genomic DNA of a donor. As suggested by Khetparal *et al.* (22) this method is favoured when P1 transduction is inapplicable, for example, when working with clinical strains on which a transducing lysate cannot be prepared. Further, this method has another application in bacterial genome engineering. Considering that in our experiments genomic DNA was sheared to approx. 30 kb, an interesting scenario develops in which electroporation favours independent transfer of closely linked mutation, which cannot be separated by P1 transduction. Furthermore, the upper limit of fragment sizes and their boundaries are easily accessible by *in vitro* treatment of DNA with rare-cutting restriction endonucleases such as LguI, SmiI, MssI and PpiI.

In this work, we demonstrated the applicability of the *cI-hok* counterselection and *ocr*-assisted recombineering strategy to four different bacteria of the family *Enterobacteriaceae*. We consider three major features to be responsible for the broad usefulness of this approach. First, the CI/P_L_/P_R_ genetic circuit is highly reliable and works well even in distant bacteria such as *Bacillus subtilis* (27,28). Second, the toxicity imposed by Hok is unlikely to be alleviated by Sok antitoxins from homologous *hok/sok* toxin-antitoxin pairs that might reside within different bacteria. This is because an active site of the Sok antisense RNA is located in the 5′-untranslated region of the *hok* transcript (29). Therefore, Sok-like antitoxins cannot alleviate Hok toxicity if *hok* is expressed under the control of a heterologous promoter. Third, Ocr exhibits activity against a wide spectrum of Type I and Type III R-M systems (64–67). We suppose that this property of Ocr might be crucial for the genome engineering of bacterial species that may possess an unknown restriction-methylation pattern. Therefore, these considerations on the design of the approach and the obtained results indicate that the proposed strategy can be a general method for engineering various microorganisms at least across the family *Enterobacteriaceae* and can potentially be adapted to even more distant taxa.

Despite the key advantages of our integration strategy, two main drawbacks should be considered. First, because of the stochastic nature of mutations and the resulting fluctuation in the frequency of mutants in a culture, some particular experiments may show an unusually high number of spontaneous mutants, thereby complicating the identification of desired recombinants. This problem could be solved by performing two parallel experiments starting with independent colonies. The second drawback is more complex. Successful selection of recombinants upon replacement of the *cI-hok* cassette requires electroporated cells to divide for several generations to ensure exhaustion of CI and derepression of *cat*. A potential obstacle may appear upon the integration of constructs encoding metabolic pathways or genetic circuits, which impose a metabolic burden that retards growth and reduces the recombinant fitness. Considering that non-recombinants grow normally, this could result in reduced apparent integration efficiency and impede the identification of edited clones. Another unfavorable scenario is the selection of correct recombinants carrying an inserted construct with point mutations that eliminate a detrimental effect on growth. Consequently, despite possessing the desired construct in the targeted locus, such recombinants are useless as the functions to be added are lost. Therefore, sequences of PCR-generated constructs to be introduced via counterselection, especially if they confer no explicit phenotype, should necessarily be verified by sequencing upon insertion into the chromosome. In this connection, one can assume that constructing an integrative cassette of interest on a plasmid vector followed by preliminary sequencing is a preferable approach. Herein, homologous arms with increased length can be introduced into the construct similar to our experiments with the *pntAB-gdhA* module. Further, when preparing the plasmid-based construct, a positive selection marker flanked by either *lox* or FRT sites can be added, thereby providing an alternative to markerless integration via counterselection. However, assuming that the exact integration site is not important, the counterselection approach still seems more attractive. Specifically, if homologous arms are designed to recombine within the preexisting dual selectable cassettes, these cassettes serve as a useful ‘landing pad’ for markerless constructs to be integrated. Thus, a single donor plasmid could be used for multiple integrations into several distinct loci and a desired mutation could be obtained in a single step, whereas the marker-based approach would require marker elimination via site-specific recombination.

In conclusion, we present a novel genome-engineering strategy combining highly restrictive counterselection and advanced recombineering, which together turn the bacterial chromosome into a general-purpose cloning vehicle and enable facile markerless cloning of large linear cassettes. This approach has substantial advantages over existing methods and can be used to expand the repertoire of tools in synthetic biology and metabolic engineering.

## DATA AVAILABILITY

The template plasmids of pTmp series (# 176227–176233), helper pRedCm (# 176225), and pRedCmOcr (# 176226) plasmids are available through AddGene. Strains with the chromosomally integrated *cI-hok* cassette and/or *ocr-γβexo*-P_L_-*cat* helper module, pRedCmOcr^SC101ts^ and pRedCmOcr^RSF^ plasmids have been deposited in the VKPM collection (Moscow, https://vkpm.genetika.ru/katalog-mikroorganizmov/).

## Supplementary Material

gkac649_Supplemental_Files

## Notes

Present address: Tigran V. Yuzbashev, Computational and Analytical Sciences Department, Rothamsted Research, Harpenden AL5 2JQ, UK.
