# Supplementary material for: Robust counterselection and advanced λRed recombineering enable markerless chromosomal integration of large heterologous constructs
Source: Nucleic Acids Res. 2022 Aug 3;50(15):8947–60. doi: 10.1093/nar/gkac649 (PMC9410887; doi:10.1093/nar/gkac649)
Supplement: gkac649_Supplemental_Files [file gkac649_supplemental_files.zip › Revised_Supplementary_Data.pdf]

SUPPLEMENTARY DATA

Robust counterselection and advanced λRed recombineering enable markerless chromosomal integration of large heterologous constructs

Dmitrii M. Bubnov[1,2,*], Tigran V. Yuzbashev[3], Andrey A. Khozov[1,2,6], Olga E. Melkina[2,5], Tatiana V. Vybornaya[1,4], Guy-Bart Stan[3], and Sergey P. Sineoky[1,2]

[1]Bioresource Center Russian National Collection of Industrial Microorganisms (BRC VKPM), State Research Institute for Genetics and Selection of Industrial Microorganisms of National Research Center «Kurchatov Institute» (NRC «Kurchatov Institute» – GosNIIgenetika), 1-st Dorozhny pr., 1, Moscow 117545, Russia

[2]Kurchatov Complex of Genetic Research, NRC «Kurchatov Institute», Kurchatov Square, 1, Moscow 123098, Russia

[3]Department of Bioengineering and Imperial College Centre for Synthetic Biology, Imperial College London, London SW7 2AZ, UK

[4]Kurchatov Genomic Center, NRC «Kurchatov Institute» - GosNIIgenetika, 1-st Dorozhny pr., 1, Moscow 117545, Russia

[5]Laboratory of Bacterial Genetics, NRC «Kurchatov Institute» – GosNIIgenetika, 1-st Dorozhny pr., 1, Moscow 117545, Russia

[6]Department of Microbiology, Faculty of Biology, Lomonosov Moscow State University, Lenin's Hills, 1-12, Moscow 119234, Russia

*To whom correspondence should be addressed. Tel: +7(495)315-07-01; Email: bubnov.dmitrii@mail.ru

Present Address: [Tigran V. Yuzbashev], Computational and Analytical Sciences Department, Rothamsted Research, Harpenden, AL5 2JQ, UK

SUPPLEMENTARY MATERIAL AND METHODS

Plasmid construction

Plasmid constructs were generated using a combination of Gibson assembly, restriction-ligation, and *in vivo* recombineering. Plasmid modification by recombineering was performed using the strain B1310 carrying defective temperature-sensitive λ prophage. λRed recombination functions were induced by heat shock followed by making the cells electrocompetent as described previously (1). The cells were co-electroporated with 100 ng of the plasmid to be modified and either 200 ng of linear dsDNA fragment or 1 µg of an oligonucleotide. For transformation of ligation and Gibson assembly reactions, homemade electrocompetent cells of DH5α or XL1 Blue strains were used. GeneJET Plasmid Miniprep Kit (Thermo Fisher Scientific Baltics, Vilnius, Lithuania) was used for plasmid DNA extraction. When plasmid was isolated from *endA*$^{+}$ strains, columns were additionally washed with 5M guanidine hydrochloride in 50% (v/v) isopropanol to avoid DNA degradation. All cloned PCR fragments and regions mutagenised by recombineering were sequenced using Sanger sequencing.

<u>Construction of pPRC</u>

The pPRC plasmid was constructed by Gibson assembly of three fragments. The fragment carrying the *rpsL* gene was amplified using primers 3050-3051 and the genomic DNA of MG1655 as the template. The second fragment included the *cat* ORF with the T(0) terminator of transcription and was amplified from the pLtet14 plasmid (2) using primers 3052-3053. The third fragment containing the *bla* marker and pSC101^{ts} origin of replication was amplified using primers 3054-3055 and pKD46 as the template. The three fragments were combined and assembled using Gibson isothermal assembly.

<u>Construction of the pTmp series of plasmids</u>

The fragment comprising the *cI*^{857}*-hok-neo* cassette was amplified using primers 3956-3957 and the genomic DNA of B1729. The sequence of the $T_{L3S1P56}$ terminator of transcription was added as part of the primer 3956. Thus, the amplified fragment carried the *cI*^{857}*-hok-neo* cassette with the terminator placed upstream of the *cI* gene C-terminus. The vector part was obtained by digestion of pMW118 (Nippon Gene, Tokyo, Japan) with SmaI and HindIII. The resulting fragment was blunt-ended with T4 polymerase. Both fragments were ligated to form pMW-$T_{L3S1P56}$-cI^{857}-hok-neo.

To correct the A67T amino acid residue substitution (C→T nucleotide substitution at coordinate 37742, GenBank Accession Number J02459.1), which causes temperature sensitivity in the CI repressor, pMW-$T_{L3S1P56}$-cI^{857}-hok-neo and oligo 3955a were co-electroporated into the

recombineering-proficient B1310 strain. Correct recombinants expressing the wild type CI repressor were selected on LB plates with kanamycin at 42°C. Cells carrying the non-recombinant plasmid were killed by the expression of plasmid-encoded *hok* and *kil* residing within the λ prophage, whose expression is repressed by CI. The resulting plasmid was named pTmpKm.

The pTmpKmHokL13Ochre plasmid is similar to pTmpKm but harbours an inactive *hok* gene allele. This plasmid was constructed in two steps. First, pMW-$T_{L3S1P56}$-cI^{857}-hok-neo and oligo 3955 were co-electroporated into B1310. Cells were allowed to recover for 1 h in 1 ml of antibiotic-free medium, and then transferred to 20 ml LB supplemented with 200 mg/l ampicillin and grown overnight. Total plasmid DNA was isolated from cells and electroporated into DH5α cells. Correct transformants carrying a plasmid with introduced *hok*^{L13ochre} mutation were selected on kanamycin-supplemented plates at 42°C. The resulting plasmid was named pMW-$T_{L3S1P56}$-cI^{857}-hok^{L13ochre}-neo. This plasmid and the oligo 3955a were then co-electroporated into B1310. The recombinant plasmid with the wild type allele of *cI* gene was selected at 42°C as B1310 cells were prevented from killing by the Kil toxin encoded by defective λ prophage within the B1310 chromosome. The resulting plasmid was named as pTmpKmHok^{L13Ochre}.

The pTmpKmH207in and pTmpKmH207out plasmids were obtained as follows. The oligonucleotides 4508 and 4509 (both 5'-phosphorylated) were annealed and ligated with pTmpKm, digested with Bsp119I, and treated with FastAp alkaline phosphatase. As both ends of the vector and insert have the same cohesive ends, the insert was ligated in two possible orientations. The recombinant plasmid carrying P_{H207} promoter directed toward the *cI-hok-neo* cassette was named pTmpKmH207in. The plasmid with the alternative orientation of the promoter was named pTmpKmH207out. The plasmids pTmpKmA1O4in and pTmpKmA1O4out were constructed in the same way but the oligonucleotides used were 4506 and 4507.

The kanamycin resistance gene of pTmpKm was replaced with two different markers. For this, *strA*, which confers streptomycin resistance, was amplified using primers 4035-4036 and RSF1010 plasmid as the template. The resulting PCR product was mixed with pTmpKm and co-electroporated into B1310 cells. Recombinant plasmids were selected on LB plates with streptomycin. The resulting plasmid was named pTmpSm. The pTmpGm was obtained in the same way but an insert comprising the gentamycin resistance gene *aacC4* was amplified using primers 4037-4038 and pOJ446 as the template.

Construction of the pRedCm series of plasmids

The pRedCm plasmid was constructed in several steps. First, pDL17 (1) was digested with MlsI and PaeI, blunt-ended by T4 polymerase treatment, and self-ligated. The resulting plasmid,

named pDL23, lost the *lacI^q*-encoding region. Thus, replication of the IPTG-sensitive origin was placed under the control of the wild-type *lacI* allele within the chromosome.

Next, pDL23 and oligo 3981 were co-electroporated into B1310 cells. Total plasmid DNA was isolated, treated with BamHI, and transformed into XL1 Blue. Successful recombination between the plasmid and oligo resulted in elimination of the unique BamHI site, thereby allowing selection of recombinant plasmid clones without any BamHI site. The recombination also caused N-terminal truncation of *gam* ORF, thereby enabling Gam translation from an internal start codon (coordinates 33112→33110, GenBank Accession Number J02459.1). Additional details regarding the alternative start-codons of Gam are described elsewhere (3, 4). The resulting plasmid was named pDL23gamS.

pRedCm was constructed by ligation of two fragments. The vector part was prepared by linearisation of pDL23gamS by NcoI digestion with T4 polymerase treatment. The insert containing *cat* under the control of the P_L promoter was amplified using oligonucleotides 4176-4177 and the genomic DNA of B1615 as the template. At this step, the ACA triplet within the -35 hexamer of the promoter (coordinates 35614→35612, GenBank Accession Number J02459.1) was randomised to achieve a lower than wild type promoter strength and chloramphenicol sensitivity of the host cell under CI-repression conditions. To select an appropriate clone, the resulting plasmid library was transformed into B1333, which expresses a temperature-sensitive CI repressor and has no toxic genes of the λ prophage. Chloramphenicol resistant transformants were selected at 42°C. These transformants were tested for sensitivity at 30°C. For this, colonies were re-isolated at 30°C to ensure Cat exhaustion. The colonies were then replica plated on LB agar with 20 mg/l chloramphenicol and plates were incubated at 30°C. Isolation and restriction analysis of plasmid DNA from chloramphenicol sensitive transformants showed that in all cases, the P_L-*cat* insert was in the same orientation as that of the $P_{T5/lacO}$ promoter, the key element dedicated for regulating plasmid replication (1). Thus, the activity of the P_L promoter may interfere with plasmid stability and lead to its elimination from host cells. To avoid this, clones selected in the previous step were re-streaked on non-selective LB agar at 42°C. Colonies were scraped from agar, resuspended and titred on non-selective LB agar and on LB agar with 200 mg/l ampicillin. A single stable clone exhibiting the same titer of cells on selective and non-selective plates was collected and named pRedCm. Subsequent Sanger sequencing revealed that the -35 hexamer of the P_L-promoter was changed from "TTGACA" to "TTGACC". The promoter also carries a single A nucleotide deletion (at coordinate 35573, GenBank Accession Number J02459.1) downstream of the transcription initiation site.

Derivatives of pRedCm carrying antirestriction functions and Red genes constituting a single artificial operon were obtained by inserting a corresponding ORF into the XbaI site of pRedCm between the P_{rhaB} promoter and *gam*. λ*ral*, *ardA* from the conjugative plasmid ColIB-P9, T7*ocr* genes were amplified using the primer pairs 4576-4577, 4578-4579, and 4580-4581 using the genomic DNA of λ phage, the plasmid p15A::ardA, and the plasmid p15A::ocr as respective templates. These PCR fragments were digested with Eco31I and XbaI, and then ligated with pRedCm linearised using XbaI. The resulting plasmids were named pRedCmRal, pRedCmArdA, and pRedCmOcr.

Construction of pBR-vioABCDE

The pBR-vioABCDE plasmid was obtained using the *in vivo* gap-repair cloning method (5). For this, the plasmid backbone comprising the pMB1 origin, tetracycline resistance marker and homology regions targeting *ΔlacZYA::vioABCDE* loci were amplified using primers 4743-4744 and pBR322 as the template. The resulting PCR product was electroporated into the recombineering-proficient strain B2193 and tetracycline-resistant colonies were isolated. Successful recombination of the PCR product leads to retrieving the targeted chromosomal region into the vector and plasmid recircularisation.

Construction of pMW-pntAB-aspC-asd-pycA-scrKYABR-gdhA

The pMW-pntAB-aspC-asd-pycA-scrKYABR-gdhA plasmid was constructed in three steps. First, a region encoding the *pntAB* operon was retrieved from the B1310 chromosome into the pMW118 vector by gap-repair cloning to produce pMW-pntAB. To do this, the vector part with homology regions targeting the *pntAB* locus was amplified using primers 4390-4391 and pMW118 as a template. The pMW-pntAB plasmid digested with ClaI and SmaI was used as a vector for the next cloning step. Inserts comprising *aspC* and *asd* genes of MG1655 were amplified with primers 4498-4499 and 4500-4501, respectively. The third insert including the *pycA* gene with an artificial promoter was synthesised using two-step PCR. First, the ORF was amplified using primers 4496-4497 and the genomic DNA of *Ensifer meliloti* ATCC 9930 as the template. The resulting fragment was re-amplified using primers 4502-4497. The vector and the three inserts were joined together using Gibson assembly to produce pMW-pntAB-aspC-asd-pycA. Finally, two inserts were cloned into pMW-pntAB-aspC-asd-pycA. The first insert is the *scrKYABR* operon, which was amplified using primers 4680-4681 and the genomic DNA of B3996. The second one, the *gdhA* gene, was amplified with primers 4682-4683 and the chromosome of MG1655 as the template. The vector part was prepared by digesting pMW-pntAB-aspC-asd-pycA

with XmaJI. The resulting fragment and both inserts were assembled using Gibson assembly to produce pMW-pntAB-aspC-asd-pycA-scrKYABR-gdhA.

Measurement of mutation rate to counterselection resistance

For the fluctuation assay, strains carrying the *cI-hok* cassette were transformed with pRedCm to enable counterselection. An overnight culture of an examined strain was inoculated into fresh LB medium without antibiotics to a final concentration of 100 cells/ml; however, for strains carrying pRedCm ampicillin was added to LB at 200 mg/l. The inoculated medium was divided into twenty 2.5 ml samples and transferred into separate 50 ml tubes. The cultures were grown overnight with shaking at 220 rpm and 37°C. For strains carrying the *cI-hok* cassette, 1 or 2 ml of culture (the same volume for each specific strain) were pelleted and spread onto LB agar containing either 200 mg/l chloramphenicol for strains carrying pRedCm or 25 mg/l chloramphenicol for strains with the *ocr-γβexo*-P_L-*cat* module within the chromosome. For strains with *rpsL* and *sacB* markers, cultures were diluted 10-fold in 0.9% sodium chloride solution and 100 µl of the diluted suspension were plated onto either LB agar with 500 mg/l streptomycin or SuLB agar. To measure the total number of viable cells, three cultures of each of the 20-sample set were diluted 10^6-fold and 100 µl were plated onto non-selective LB plates or on LB plates with 200 mg/l ampicillin for strains carrying pRedCm. After 24 h of incubation at 37°C, the mutants that survived counterselection were counted. The average of three independent determinations of the viable cell titre was used for all twenty samples within each set. Mutation rate was calculated using the Ma-Sandri-Sarkar maximum likelihood method (6), and the confidence interval was calculated as described previously (7). The calculations were performed using FluCalc software (8). If several samples within a particular set contained no mutants and a ratio of such samples was in the range of 0.1–0.7, the *p0*-method of Luria and Delbruck (9) was used to calculate the mean number of mutations per culture (m). The mutation rate (μ) was calculated by dividing m by the total number of cells divisions, which is assumed to be numerically equal to the number of plated cells (7). As only a fraction of the cultures was plated, the mutation rate was corrected by the estimated plating efficiency. The 95% confidence limits were calculated using F statistics. Both these calculations were performed as described by Foster (7).

Assay of λ phage plating efficiency

The stock of the λ bacteriophage (λ*cI857,* deposited in VKPM as B-7373) was prepared using the lysogenic strain B1270. Cells were grown overnight in LB at 30°C. The culture was diluted 200-fold in the same medium and allowed to grow at a permissive temperature until the OD_{600} reached 0.3–0.4. The cells were then transferred to a shaking water bath and incubated at 42°C

for 2 h. A 1 ml aliquot was mixed with 100 µl of chloroform, vortexed, and centrifuged at 12000 × *g*. The upper phase was collected and stored at -4°C. The stock of unmethylated λ phage (referred to as λ.0) was prepared by infecting the strain TG1, which lacks both EcoKI endonuclease and methyltransferase activity.

The phage titre was determined by plating on a lawn of the host strain, and counting the resulting plaques. Serial 10-fold dilutions of the phage stock were prepared in 0.9% sodium chloride solution. A 100-µl aliquot of phage suspension was mixed with 200 µl of a host cell culture. The mixture was added to 5 ml of melted top agar (LB with 0.75% agar) at 45°C and spread over a prewarmed LB plate. The plates were incubated overnight at 37°C and then counted. Host cells were grown in the same way used for competent cell preparation. Experiments for determining λ and λ.0 plating efficiency on cells carrying the pRedCm, pRedCmRal, pRedCmArdA, or pRedCmOcr plasmids were performed in parallel with the recombineering experiment. Therefore, the same cells induced for expressing antirestriction functions and the Red operon were used in both assays. The aliquot for the measurement of plating efficiency was collected immediately once induction was completed and cells were chilled in an ice water bath.

Measurement of *in vivo* luminescence and bacterial growth

A single colony of an assayed strain was inoculated into 5 ml of LB without any supplements. The overnight culture was diluted to a starting OD_{600} of 0.004 in fresh LB. If necessary, 1 mM IPTG was supplemented at zero time point for inducing LacI-repressible promoters. This culture was transferred to a 96-well plate at 200 µl of culture/well (black-walled, transparent flat bottom; cat. #665096 Greiner Bio-One, Frickenhausen, Germany). The outer wells were not used to avoid edge effects. For each strain, 6 wells were used as technical replicates. Strains were ordered across the plate according to promoter strength from the highest to lowest for reduced luminescent cross-talk. A well with sterile LB was set as the blank. The plates were incubated at 37°C with double-orbital shaking at 600 rpm using CLARIOstar Plus luminometer (BMG Labtech, Ortenberg, Germany). OD_{600} and luminescence were measured every 15 min. No luminescence emission filter was applied. The photomultiplier gain was automatically controlled by the enhanced dynamic range function. Measured values were normalised to 1 s accumulation time.

Acquired data were analysed using MARS software. Blank values were subtracted from raw OD_{600} and relative luminescence units (RLU) values. Corrected RLU reads at each time point were divided by the corresponding OD_{600} values to normalise RLU per cell mass for each well. The average RLU/OD_{600} values and standard deviations were calculated and plotted against time. For

calculating the specific growth rate (μ) of a strain, the natural logarithm of OD_{600} was plotted against time. An argument range where the logarithm exhibits linear dependence on time was used for fitting by employing the linear regression fit model. The μ value (h^{-1}) was found as a slope of the resulting curve. Doubling time (t, min) was calculated using the following formula:

$$t = \frac{60 \cdot \ln 2}{\mu}$$

Preparation of linear cassettes

Except the *vioABCDE* and *pntAB-gdhA* cassettes, linear DNA cassettes were generated by PCR using Kapa HiFi DNA polymerase with homology arms targeting a defined locus within the chromosome. The length of homology arms was either 50 bp for the *cat-sacB, rpsL-cat*, and *cI-hok* dual selectable cassettes, or 72–82 bp for the markerless cassettes designed to be inserted via *cI-hok* counterselection. The complete sequences of cassettes, primers, and templates used for amplification are listed in Supplementary Table S4. The *vio* cassette was generated as follows. The plasmid pBR-vioABCDE was isolated from the non-methylating strain TG1, and digested with PdmI and Bst1107I followed by FastAp alkaline phosphatase treatment, thereby generating a linear fragment of 7525 bp in length with blunt nonphosphorylated ends. The *vioABCDE* operon within this fragment is flanked by homology regions of 72 bp and 80 bp in length targeting the P_{lacZYA} promoter and region downstream of the *lacA* gene C-terminus, respectively. The *pntAB-gdhA* cassette was prepared from the pMW-pntAB-aspC-asd-pycA-scrKYABR-gdhA plasmid. Plasmid DNA was isolated from XL1 Blue cells and digested using Eam1105I and PscI. The linear fragment was blunt-ended by T4 polymerase treatment. The resulting *pntAB-gdhA* cassette was 21971 bp in length with homology arms of 998 bp and 898 bp in length, which targeted regions upstream of *cI* and downstream of an antibiotic resistance gene within plasmids of the pTmp series.

PCR-generated cassettes were precipitated with ethanol and digested using DpnI to eliminate methylated template DNA. Dual selectable cassettes were purified using Monarch PCR & DNA cleanup kit (New England Biolabs, Ipswich, USA) omitting the gel-purification step. The manufacturer’s instructions were followed with two minor modifications. First, ice-cold 80% ethanol was used instead of the included wash buffer for the second washing step. Second, DNA was dissolved in deionised water. These modifications allowed elution of highly desalted DNA, which favoured efficient electroporation. Markerless cassettes were processed similarly; however, after DpnI treatment this DNA was thoroughly purified using preparative gel-electrophoresis. To avoid DNA damage, longwave UV light (365 nm) was used during

preparations. DNA was extracted from agarose gel using the GeneJET Gel Extraction Kit (Thermo Fisher Scientific Baltics, Vilnius, Lithuania). The resulting DNA was resolved in an analytical gel and examined for the presence of impurities. If any visible contamination was detected, gel-purification was repeated. As nonspecific amplicons had the same flanking homology arms as the desired PCR fragment, they competed with it for recombination in the targeted locus. Special care should be taken when purifying short constructs (shorter than 1–2 kb) from PCR primers and their dimers as they migrate close to each other in the agarose gel. The dimers could be a direct substrate for recombination and compete with the desired construct. Primers also could recombine effectively via the Red pathway if they have even a limited homology of 6 bp on their 3'-ends (10). Therefore, it is essential to use DNA of the highest possible purity for markerless replacement of the counterselectable cassette.

Purified DNA was concentrated using the Monarch PCR & DNA cleanup kit (New England Biolabs, Ipswich, USA) as aforementioned to obtain a final concentration of 0.2–0.5 µg/µL. Plasmid-derived markerless cassettes were purified in the same way used for PCR-generated DNA fragments except for DpnI treatment. DNA was quantified using gel electrophoresis and ethidium bromide staining with the GeneRuler 1 kb DNA Ladder (Thermo Fisher Scientific Baltics, Vilnius, Lithuania) as a standard. Aliquots were stored at -20°C.

We note that in our experiments, markerless cassettes designed to be inserted via *cI-hok* counterselection were flanked with 80-bp homology arms instead of the regular 50-bp arms. Does the longer homology ensure a significant difference in recombination efficiency, thereby increasing the reliability of cloning constructs through counterselection? To answer this question we prepared two sets of the *scrKYABR* cassettes targeting the *galETKM* and *araBAD* operons and compared their recombination efficiencies using the MG1655 strain and pRedCmOcr helper. Recombinants were positively selected on sucrose-supplemented M9 agar. As seen from Supplementary Figure S14 the cassettes flanked by the 80-bp arms generally recombined better.

On the other hand, the same results indicate that the recombination frequencies with cassettes targeting the *ara* locus are significantly lower than those of the *gal::scr* cassettes. However, when introducing the *scr* cassettes into these two loci using *cI-hok* counterselection we observed (Figure 4C) nearly the same percentage of positive recombinants (~100% and 90% for the *gal* and *ara* locus, respectively) (Figure 4 in the main manuscript). Notably, the sequence of the *scr* cassettes with 80-bp homology arms used for the experiments, presented in Supplementary Figure S14 and Figure 4C, is the same. This means that even the lower recombination efficiency observed in the *ara* locus is sufficient to successfully insert the *scr*

cassette using *cI-hok* counterselection. Thus, cassettes with homologous arms shorter than 80 bp might be usable. However, we did not examine this possibility. Further, we cannot exclude that some cassettes in some loci can recombine so poorly that it is difficult to isolate the desired recombinants when using 50 bp homology. In this case, slight enlargement of the arms to 80-bp would be crucial. Therefore, we suggest that homology of 80 bp for operon-size cassettes is a good compromise between the reliability of integration and the cost of PCR primers.

Preparation of electrocompetent cells

A single colony of an appropriate strain was picked and inoculated into 5 ml LB. Strains carrying λRed-expressing helper plasmids were grown in ampicillin-supplemented LB medium. In experiments for studying the efficiency of *cI-hok* replacement with heterologous DNA, strains were grown overnight in the presence of gentamycin, kanamycin, or streptomycin at concentrations described in the section Media and culture conditions to avoid spontaneous elimination of dual selectable cassettes. The overnight culture was diluted 200-fold in 10 ml of LB media. At this step, the concentrations of ampicillin, gentamycin, kanamycin, and streptomycin were reduced to 100 mg/l, 5 mg/l, 25 mg/l, and 5 mg/l, respectively. The cultures were incubated in 50 ml test tubes until OD_{600} reached 0.3–0.4 (for 100–110 min). Strains harbouring pDL17, pRedCm, pRedCmRal, pRedCmArdA, or pRedCmOcr were induced for expressing λRed functions by adding 200 µL of prewarmed 20% (w/v) L-rhamnose solution. Strains with the chromosomally integrated P_{H207}-*lacI*-$P_{A1lacO\text{-}1}$-*ocr-γβexo*-P_L-*cat* module were induced using 2 mM IPTG. In both cases, cells were incubated for another 25 min and placed in an ice-water bath. All subsequent steps were performed on ice. Cells were pelleted at 8000 × *g* for 2 min at 4°C in 50 ml polypropylene tubes. The supernatant was discarded, the tubes were centrifuged for another 20 s and the residual medium was aspirated. Cells were gently resuspended and washed once with 35 ml of sterile ice-cold deionised water. The pellet was then resuspended in 1 ml of water and transferred to a precooled 1.5 ml tube. The tube was centrifuged for 45 s in a cooled microcentrifuge rotor at 12000 × *g*, the supernatant was thoroughly aspirated. The cell pellet was resuspended in 80 µL of ice-cold deionised sterile water and used for electroporation within 1 h. In all recombineering experiments only freshly prepared electrocompetent cells were used.

Helper plasmid curing

To eliminate the helper pRedCm or pRedCmOcr plasmids, colonies were re-steaked on LB plates with 1 mM IPTG. Under these conditions, plasmid replication is inhibited. The resulting colonies mostly comprise plasmid-free cells, but still include some cells that retain the non-

replicating plasmid. These colonies were re-purified a second time on LB agar without supplements and the resulting colonies were tested for ampicillin sensitivity.

Markerless transfer of genomic loci by P1 transduction or transformation with genomic DNA

Strains with the *cI-hok* cassette inserted into the *gal, lac, ara* or *man* locus were used as recipients for P1 transduction. The P_L-*cat* module was provided on the pRedCmOcr plasmid or chromosomal helper module to enable *cI-hok* counterselection. Successful transfer of wild-type loci from MG1655 into these strains restored the ability to utilise the corresponding carbohydrate and enabled discrimination of recombinants.

A single colony of a recipient strain was picked into 5 ml of LB broth supplemented with ampicillin (200 mg/l) for maintenance of pRedCmOcr along with kanamycin (100 mg/l), gentamycin (20 mg/l), or streptomycin (20 mg/l) as needed. On the next day, overnight cultures were diluted 100-fold in 10 ml of LB with the same antibiotics, except for ampicillin whose concentration was reduced to 100 mg/L. Incubation proceeded until OD_{600} reached 0.6–0.8. Cells were harvested by centrifugation, resuspended in 2 ml of MC buffer (100 mM $MgSO_4$, 5 mM $CaCl_2$) in a 50 ml centrifugation tube and incubated for 20 min with shaking at 220 rpm and room temperature. A 200 µl aliquot of the suspension was transferred to a 1.5 ml polypropylene tube and mixed with 0.5×10^8–1.0×10^8 pfu of P1*vir* lysate prepared using the plate method (11). The mixture was incubated for 20 min at 37°C with shaking at 1000 rpm. Infection was stopped by transferring the suspension into 20 ml LB with 167 mM sodium citrate. Following overnight growth, cells were plated onto LB agar with either 200 mg/l or 25 mg/l chloramphenicol depending on whether pRedCmOcr or the chromosomal module were used as the helper. Selective plates were additionally supplemented with 25 mg/l 2,3,5-triphenyltetrazolium chloride and 1% of the appropriate carbohydrate for screening Gal^+, Ara^+, and Man^+ recombinants along with 2 mM sodium citrate to avoid phage adsorption. Lactose interferes with pRedCmOcr replication and induces toxic expression of the *ocr-γβexo* operon within the chromosomal helper. Therefore, transduction reactions of B1827 and B2141 strains were first plated on chloramphenicol agar with citrate, and colonies were then randomly picked and replica plated onto tetrazolium indicator plates with 1% lactose to discriminate the recombinants. The medium for B2141 was additionally supplemented with 1 mM IPTG as elevated LacI synthesis in B2141 renders the *lac* operon partially insensitive to lactose.

Alternatively, wild-type loci were restored by electroporation of fragmented genomic DNA from a donor strain. For this, genomic DNA was prepared using the GeneJet Genomic DNA purification kit (Thermo Fisher Scientific Baltics, Vilnius, Lithuania), which according to the

manufacturer's manual, provides DNA that is sheared to approximately 30 kb fragments. The resulting DNA was then concentrated using Monarch PCR & DNA cleanup kit (New England Biolabs, Ipswich, USA) to approximately 0.4–0.5 µg/µl. Before DNA elution with deionised water, the column was washed with ice-cold 70% ethanol. The recipient strains were induced to express λRed and made electrocompetent as described under "Preparation of electrocompetent cells" in the Supplementary Material and Methods. Cells were transformed with 2–2.5 µg of genomic DNA. Subsequent recovery, plating, selection, and identification of recombinants was performed as described for the transduction experiments, except the media was not supplemented with citrate.

Recombineering and *cI-hok* counterselection in *Salmonella*, *Citrobacter*, and *Pantoea*

The protocols for both recombineering and *cI-hok* counterselection in *Salmonella*, *Citrobacter*, and *Pantoea* were similar to those described in the sections "λRed recombineering" and "*cI-hok* counterselection and identification of recombinants" in the Materials and Methods of the main manuscript and the section "Preparation of electrocompetent cells" in the Supplementary Material and Methods with only minor modifications, which are summarized in Supplementary Table S8. The most important modification is the employment of different helper plasmids. Specifically, in the case of *Salmonella* and *Pantoea*, pRedCmOcr^{SC101ts} and pRedCmOcr^{RSF} were respectively used to supply cells with the λRed, Ocr, and CI-repressible Cat functions. In *Citrobacter* cells, the regular pRedCmOcr helper was found to work well.

GenBank records NC_003197.2, NZ_CP049015, and AP012032.2 were used as reference genome sequences for *Salmonella typhimurium* LT2, *Citrobacter freundii* ATCC 8090, and *Pantoea ananatis* SC17(0), respectively.

Supplementary Table S8. Protocol modifications for *Salmonella*, *Citrobacter*, and *Pantoea*

	**Bacterium**		
**Protocol details**	***Salmonella typhimurium* LT2**	***Citrobacter freundii* ATCC 8090**	***Pantoea ananatis* SC17(0)**
**General manipulations**			
Growth temperature	37 °C	37 °C	30 °C
Medium for washing competent cells	Deionized water	Deionized water	10% Glycerol
**Transformation with a helper plasmid**			
Helper plasmid	pRedCmOcr^{SC101ts}	pRedCmOcr	pRedCmOcr^{RSF}
Medium used to select transformants	LB plus 200 mg/l ampicillin	LB plus 200 mg/l ampicillin	LB plus 20 mg/l streptomycin
Growth temperature for competent cell preparations	37 °C	30 °C (because *Citrobacter* cells form	30 °C

		aggregates in liquid LB medium at 37 °C)	
**Integration of the *cI-hok-neo* cassette**			
Medium for an overnight culture used as an inoculum for competent cell preparation	LB plus 100 mg/l ampicillin	LB plus 100 mg/l ampicillin	LB plus 20 mg/l streptomycin
Growth temperature for competent cells preparations	30 °C (because of the temperature-sensitive pSC101^{ts} ori)	30 °C (because *Citrobacter* cells form aggregates is liquid LB medium at 37°C)	30 °C
Medium for the growth of competent cells	LB plus 50 mg/l ampicillin	LB plus 50 mg/l ampicillin	LB plus 20 mg/l streptomycin
λRed induction conditions	2 mM IPTG for 30 min	0.4% L-rhamnose for 30 min	2 mM IPTG for 40–50 min
Growth conditions used for selection of recombinants carrying the *cI-hok-neo* cassette	30 °C, LB plus 50 mg/l kanamycin	37 °C, LB plus 50 mg/l kanamycin	30 °C, LB plus 25 mg/l kanamycin
**Replacement of the *cI-hok-neo* cassette with a markerless cassette via counterselection**			
Medium for an overnight culture used as an inoculum for competent cell preparation	LB plus 100 mg/l ampicillin and 50 mg/l kanamycin	LB plus 100 mg/l ampicillin and 50 mg/l kanamycin	LB plus 20 mg/l streptomycin and 25 mg/l kanamycin
Medium for the growth of competent cells	LB plus 50 mg/l ampicillin and 25 mg/l kanamycin	LB plus 50 mg/l ampicillin and 25 mg/l kanamycin	LB plus 20 mg/l streptomycin and 25 mg/l kanamycin
Growth conditions used for the *cI-hok* counterselection	30 °C; LB plus 100 mg/l chloramphenicol supplemented with 25 mg/l triphenyltetrazolium chloride and 1% of an appropriate carbohydrate	37 °C; LB plus 200 mg/l chloramphenicol supplemented with 25 mg/l triphenyltetrazolium chloride and 1% of an appropriate carbohydrate	30 °C; LB plus 100 mg/l chloramphenicol supplemented with 25 mg/l triphenyltetrazolium chloride and 1% of an appropriate carbohydrate

Estimating the capacity of strains for amino acid accumulation

To examine amino acid accumulation, strains of interest were inoculated using a sterile toothpick in a 50-ml test tube containing 2 ml of a seed medium with the following composition (g/l): yeast extract, 35; K_2HPO_4, 2.5; glucose, 2.5; NaCl, 2.5; initial pH 7.2. The seed cultures were grown overnight at 37 °C and 220 rpm. The next day, a 100 µl aliquot of the seed culture was

inoculated in a 50-ml test tube with 2 ml of fermentation medium (g/l): $(NH_4)_2SO_4$, 30; K_2HPO_4, 2.5; citric acid, 0.192; corn steep liquor (Roquette, Lestrem, France), 10; $FeSO_4 \cdot 7H_2O$, 0.03; $MnSO_4 \cdot H_2O$, 0.02; glucose, 40; $MgSO_4 \cdot 7H_2O$, 2; $CaCO_3$, 20; initial pH 7.2. The inoculated test tubes were incubated for 24 hours at 37 °C and 220 rpm. Cells were then precipitated by centrifugation for 5 minutes at 13000 × *g*. The supernatant was diluted 3-fold with deionized water and analyzed for amino acid content using HPLC. HPLC analysis was performed using a Waters Alliance 2695 HPLC system equipped with YMC-Pack Polyamine II columns (YMC, Kyoto, Japan) and a mixture of ethyl acetate:acetonitrile:H_2O (4:46:50) as the mobile phase. The concentration of L-threonine and L-tryptophan was determined using a Waters 2414 refractometer at 420 nm.

SUPPLEMENTARY RESULTS

1. The $T_{L3S1P56}$ terminator protects the *cI-hok* cassette from surrounding promoters

Using the strong P_{A1O4} promoter (12) we verified that the $T_{L3S1P56}$ terminator upstream of the *cI-hok* cassette reliably protects *hok* from neighbouring transcription signals. For this, we generated the B2096 strain with P_{A1O4}-*cI-hok-neo* inserted in front of the promoterless *luxCDABE* operon of *Photorhabdus luminescence* (Supplementary Figure S1). If transcription from P_{A1O4} proceeds through the cassette to the *lux* operon, we would observe luminescence. Indeed, measurement of *in vivo* luminescence showed that P_{A1O4} causes low-level expression of *luxCDABE*. However, the luminescence of this strain exceeds that of the negative control strain MG1655 by approximately 2-fold, whereas insertion of P_{A1O4} directly upstream of the reporter construct increases luminescence 10^5 times over the background level. Additionally, *hok* derepression caused by transcriptional readthrough can potentially lead to reduction in the growth rate. We measured the doubling time of the strain carrying the P_{A1O4}-*cI-hok-neo* in LB and did not find significant differences compared to that of wild-type strain MG1655. Considering that P_{A1O4} is one of the strongest promoters in *E. coli*, these results indicate that *cI-hok* cassettes could be inserted anywhere around the genome without being affected by resident promoters.

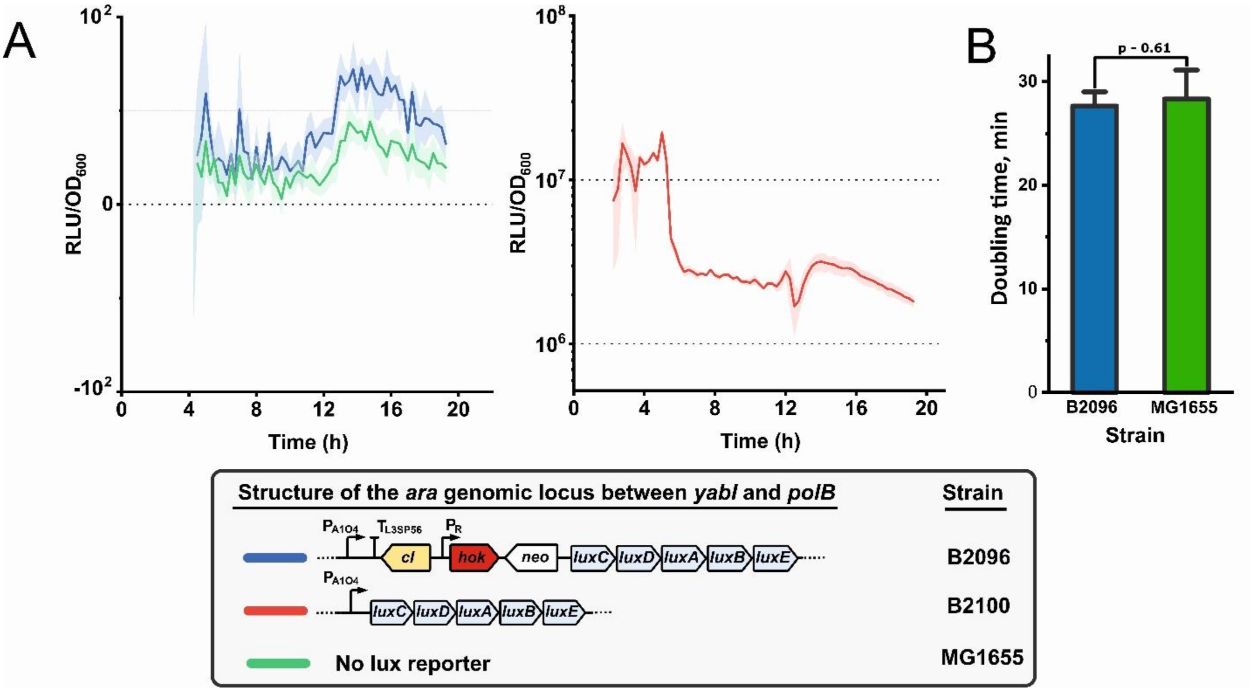

Supplementary Figure S1. The $T_{L3S1P56}$ terminator protects the *cI-hok* cassette from surrounding promoters.

(A) The lux reporter output of the P_{A1O4}-*cI-hok-neo* cassette or the P_{A1O4} promoter fused to the *luxCDABE* operon. In the first case, the $T_{L3S1P56}$ terminator resides between P_{A1O4} and *luxCDABE*. The plotted data show a time course of luminescence measurements normalised to OD_{600}, corresponding to growth phases from the early logarithmic (OD_{600}

0.1–0.2) to late stationary phase (OD_{600} 1.7–1.9). Early time points corresponding to OD600 less than 0.1 were trimmed. Measurement was performed as described in the section "Measurement of *in vivo* luminescence and bacterial growth" of Supplementary Materials and Methods. The seed culture was grown overnight without induction. Upon dilution in fresh LB (at zero time point of the represented experiment) the culture was supplemented with 1 mM IPTG to induce the P_{A1O4} promoter. The data for the non-luminescent MG1655 strain serves as an indicator for the background luminescence level. The data represent the average of 6 technical replicates (6 wells inoculated from the same overnight culture), shadowed areas indicate SD. (B) Effect of the P_{A1O4} promoter upstream of the *cI-hok* cassette on the growth rate. The doubling time in the logarithmic growth phase is shown. Growth measurements correspond to the experiments shown in Figure S1A. For the B2096 strain, the medium was supplemented with 1 mM IPTG. The data represent the average of 6 technical replicates (6 wells inoculated from the same overnight culture), error bars indicate SD. The values were compared using t-test with unequal variances (two-sided p-value = 0.61).

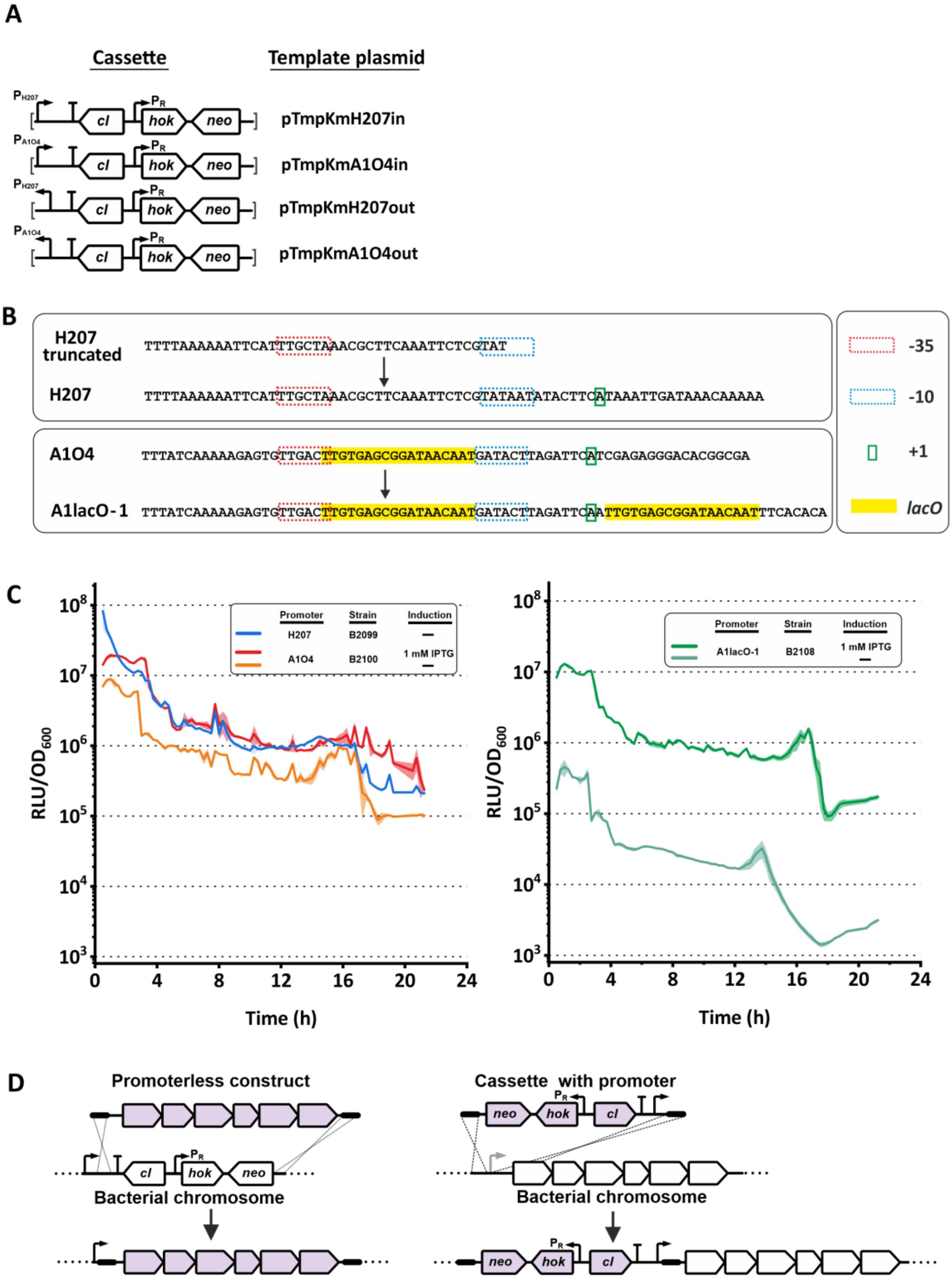

Supplementary Figure S2. The *cI-hok* cassettes with the adjacent P_{H207} and P_{A1O4} promoters. (A) List of template constructs. For each promoter, there are two alternative plasmids in which the promoter is directed toward or outward from the $T_{L3S1P56}$ terminator. The complete sequence of the template plasmids is listed in Supplementary Table S3. (B) Sequence of the P_{H207} (top panel) and P_{A1O4} (bottom panel) promoters within the template plasmids. We anticipated that P_{H207} could cause *hok* derepression even in presence of the strong $T_{L3S1P56}$ terminator between them. Therefore, we cloned P_{H207} in a truncated form lacking the last three nucleotides of the -10 hexamer and the transcription start site, which could be restored by adding them to a PCR primer. Although P_{A1O4} is known as a LacI-repressible promoter (12), its repression is weak in the wild-type genetic background. Using lux-reporter we observed approximately 2–5-fold repression in LB medium without IPTG (see Supplementary Figure S2C). However,

P_{A1O4} could be easily converted into $P_{A1lacO-1}$ (13) by adding a second *lac*-operator upon cassette amplification. This promoter exhibited 20–60-fold repression in the wild-type background (Supplementary Figure S2C) and approximately 10^3-fold repression at an elevated level of LacI (Supplementary Figure S7A). (C) The lux reporter output of P_{H207}, P_{A1O4}, and $P_{A1lacO-1}$ promoter fusions to the *luxCDABE* operon. The plotted data show a time course of luminescence measurements normalised to OD_{600}, corresponding to growth phases from the early logarithmic (OD_{600} 0.05-0.1) to late stationary phase (OD_{600} 1.7–1.9). Early time points corresponding to OD600 less than 0.05 were trimmed. Measurement was performed as described in "Measurement of *in vivo* luminescence and bacterial growth" in Supplementary Materials and Methods. The seed culture was grown overnight without induction. Upon dilution in fresh LB (at zero time point of the represented experiment) the culture was supplemented with 1 mM IPTG to induce the P_{A1O4} and $P_{A1lacO-1}$ promoters. The data represent the average of 6 technical replicates (6 wells inoculated from the same overnight culture), shadowed areas indicate SD. (D) Two scenarios of application of the promoter-carrying cassettes. The cassette with a promotor directed toward the $T_{L3S1P56}$ terminator was first inserted into the bacterial chromosome. Then, it could be replaced by a promoterless construct (indicated in violet), whereby it would be placed under the control of the heterologous promoter. Cassettes with a promoter in reverse orientation could be directly used for replacing the native promoters.

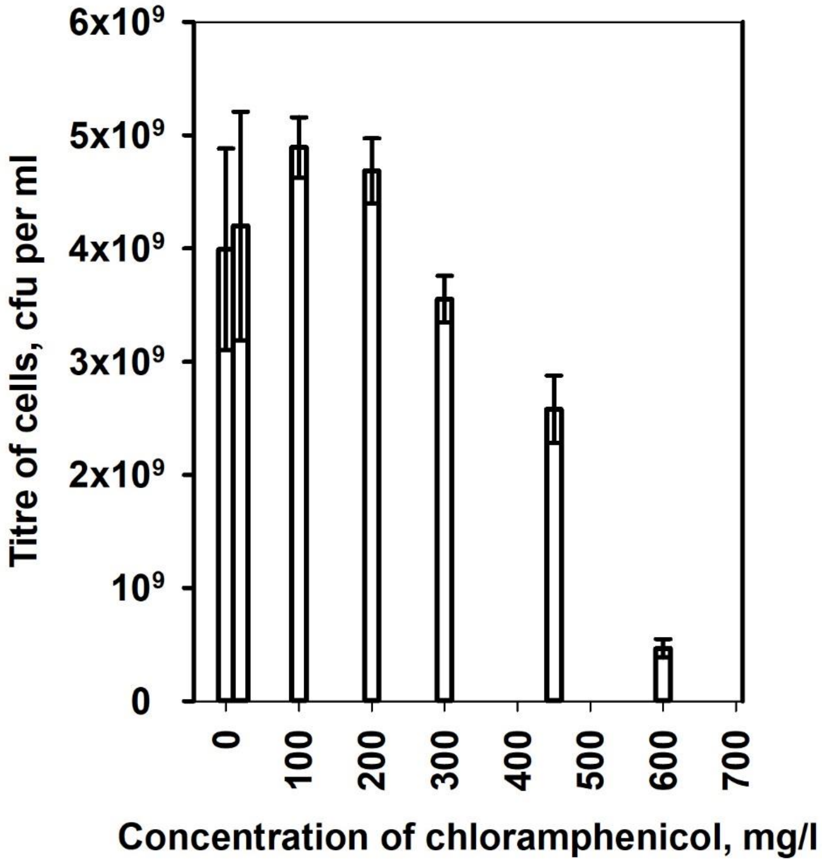

Supplementary Figure S3. The effect of chloramphenicol concentration in LB on the plating efficiency of the MG655 strain carrying pRedCm. An overnight culture grown in LB plus ampicillin (200 mg/l) was diluted 10^6-fold and plated on LB agar supplemented with ampicillin alone (200 mg/l), or with ampicillin plus chloramphenicol. The plates were incubated for 24 h and colonies were then counted. The values shown are the average of three biological replicates; error bars indicate SD.

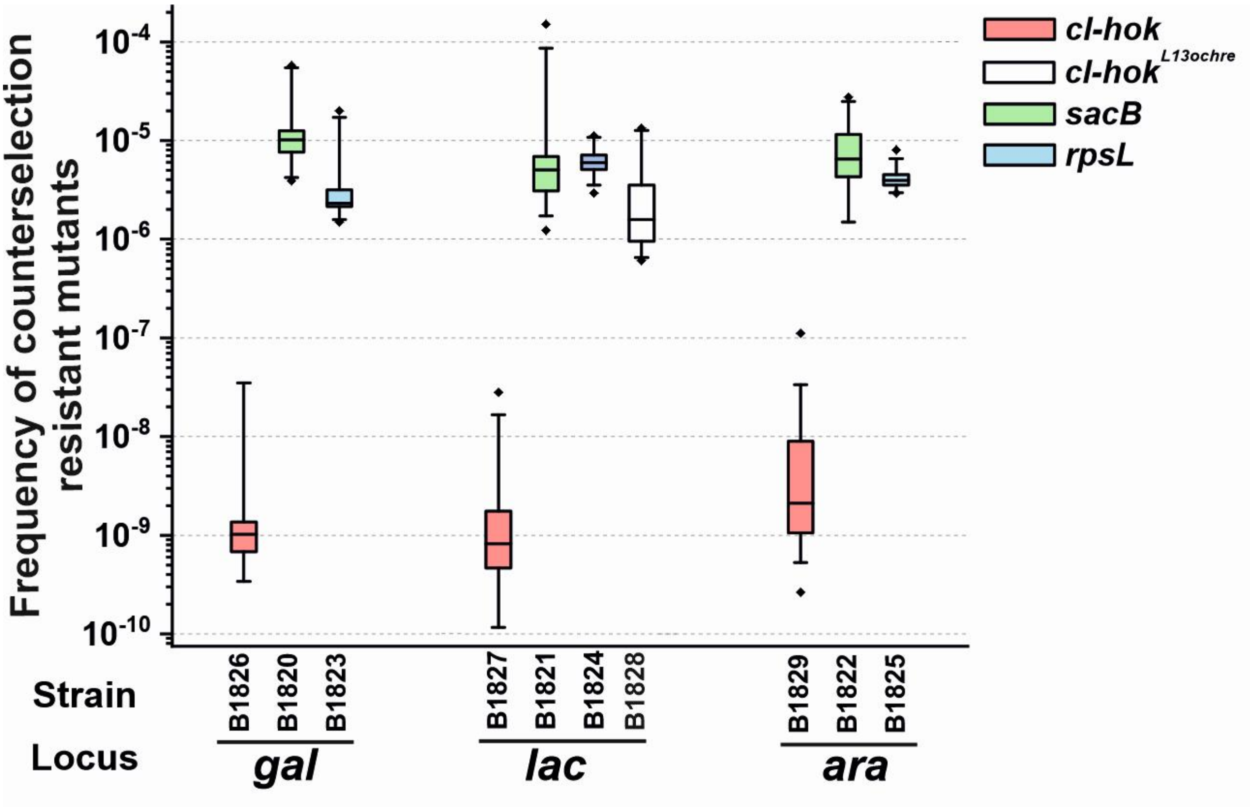

Supplementary Figure S4. Frequency of mutants resistant to counterselection for the *cI-hok, cI-hok*^{L13UAA}*, sacB*, and *rpsL* markers. Strains with *cI-hok* cassettes harboured the pRedCm plasmid. Briefly, 20 independent overnight cultures for each strain were plated onto corresponding selective plates (LB plus chloramphenicol 200 mg/l, SuLB, and LB plus streptomycin 500 mg/l). Three cultures for each strain were diluted 10^6-fold and plated onto non-selective LB agar for determining the total titre of viable cells. The frequency values were calculated by dividing the determined titre of mutants by the average titre of viable cells. Data are represented as a box-plot where the upper and lower bounds of the box are the first and third quartile; the horizontal line inside the box is the median; whiskers indicate the 5% and 95% quantiles; the points outside the whiskers are outliers. As seven cultures were found to contain no Cm^R mutants in the experiment with the B1826 strain, the box plot was constructed based only on 13 non-zero values out of 20 total values.

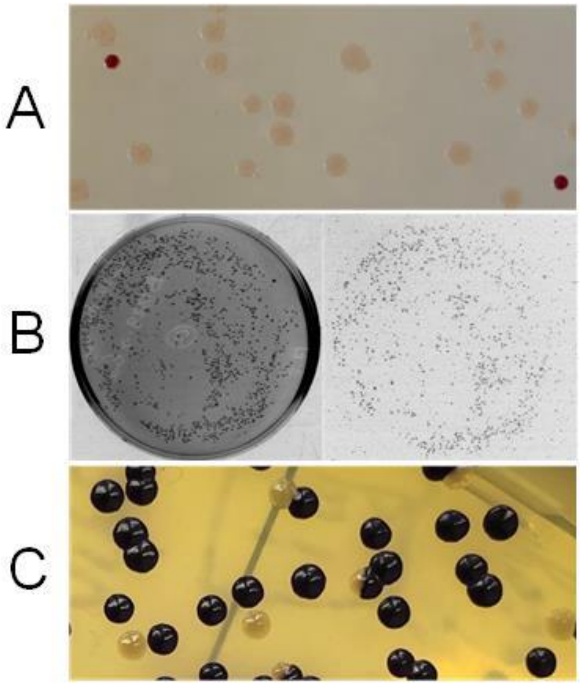

Supplementary Figure S5. Phenotypes conferred by the *scrKYABR* (A), *luxCDABE* (B), and *vioABCDE* (C) operons in *cI-hok* cassette replacement experiments. (A) A photograph of a plate on which cells of the B2141 strain electroporated with the *lac::scrKYABR* cassette were spread. Sucrose-utilizing Scr^{+} recombinants (colourless colonies) were selected on tetrazolium agar plus 1% sucrose and 25 mg/l chloramphenicol. Red colonies are Scr^{-} spontaneous Cm^{R} mutants. (B) A photograph of a plate on which cells of the B1829[pRedCm] strain electroporated with the *ara::luxCDABE* cassette were spread. Luminescent recombinants were selected on LB agar plus 0.2% L-arabinose and 200 mg/l chloramphenicol. The left photograph was captured using the GelDoc Gel Documentation System (BioRad) under white-light illumination; the right one was captured with an exposure time of 300 s in the dark. (C) A photograph of a plate on which cells of the B1827[pRedCmOcr] strain electroporated with the *lac::vioABCDE* cassette were spread. Violacein-producing recombinants (violet colonies) were selected on LB agar plus 200 mg/l chloramphenicol and incubated for 2 days at room temperature until recombinant colonies turned deep violet. Colourless colonies are spontaneous Cm^{R} mutants.

2. Analysis of spontaneous mutants that survive *cI-hok* counterselection

Using the pRedCm helper we isolated 32 independent spontaneous mutants for each of the three B1826, B1827, and B1897 strains harbouring the *cI-hok* cassette within the *gal, lac,* and *ara* locus, respectively. Following mutant purification on selective plates, we examined the structure of respective genomic loci using PCR with primers, which anneal upstream and downstream of the *cI-hok* cassette (Supplementary Figure S6A). We found that PCR from some mutants yielded a fragment markedly shorter than that corresponding to the intact *cI-hok* cassette or yielded no fragment at all (Supplementary Figure S6B). We hypothesised that the first type of mutants lost some sequence between the priming sites, whereas the second type lost one or two priming sites as a result of spontaneous deletion. To validate this assumption, we tested these mutants for resistance to the respective antibiotics (streptomycin, kanamycin, or gentamycin). Most mutants, which were assumed to carry deletions according to the PCR results, were also sensitive to the antibiotic (Supplementary Figure S6B, left panel). There were 15, 9, and 14 such mutants among the 32 tested in the *gal-, lac-,* and *ara*-group, respectively. Two mutants, one in the *ara*-group and one in the *lac*-group, yielded a visibly shorter fragment than expected but retained resistance. Apparently, in these clones, a deletion involved both the *cI* and *hok* genes but not the positive selection marker.

To further validate that a portion of spontaneous Cm^R mutants arises due to a deletion encompassing the *cI-hok* cassette we examined the selected mutants for a phenotype of genomic loci adjacent to the site of *cI-hok* cassette insertion. Inspection of the genetic map revealed that the *gal* locus is closely linked to the *pgl* gene, and that the *lac* and *ara* loci reside near the *prpBCDE* and *leuABCD* operons, respectively (Supplementary Figure S6C). Owing to a lack of 6-phosphogluconolactonase activity, disruption of *pgl* induces a Blu phenotype (14), indicated by dark-blue staining upon exposure to iodine vapour, which is caused by starch accumulation in cells grown on maltose minimal media (Supplementary Figure S6D). The *leuABCD* and *prpBCDE* operons encode the leucine biosynthesis and propionate utilisation pathway. Considering the lack of essential genes in intervals between the loci for each of the *gal-pgl*, *lac-prp*, and *ara-leu* pairs, we anticipated that some selected mutants could lose the linked chromosomal region along with the *cI-hok* cassette. Indeed, 4 mutants of 15 from the *gal*-group (those that lost streptomycin resistance) also exhibited the Blu phenotype (see the right panel of Supplementary Figure S6B). None of these mutants yielded any PCR fragment. This is in agreement with the hypothesis that these mutants emerged through a deletion encompassing the entire region between *cI-hok* and *pgl.* We then examined mutants of the *lac-* and *ara*-group in the same

manner. We found that 4 kanamycin-sensitive mutants from the first group were unable to grow on propionic acid as a single carbon source, and that 3 gentamicin-sensitive mutants from the second group were leucine auxotrophs. This indicates that deletions in these mutants extend from the *cI-hok* cassette to *prpBCDE* and *leuABCD*, respectively.

We hypothesised that other mutants retaining the intact *cI-hok* cassette regained resistance to chloramphenicol owing to mutations of the pRedCm plasmid. To test this, we isolated plasmid DNA from 12 spontaneous mutants (four mutants that retained the *cI-hok* cassette and the positive selection marker from each group) and retransformed it into the B1826 strain. All plasmids except one yielded colonies resistant to both chloramphenicol and ampicillin, indicating that chloramphenicol resistance of the original mutants was conferred by a mutation of the plasmid. Analysis by restriction digestion and sequencing revealed that 10 of 11 remaining plasmids arose from a duplication of *cat* so that the second copy was placed directly downstream of the $P_{T5/\mathit{lacO}}$ promoter (Supplementary Figure S6E and F). In these mutants, *cat* appeared to be expressed because of the basal activity of $P_{T5/lacO}$. Two remaining mutants were considered as a minor class and were not analysed further.

Taken together, these results indicate that there are two major possible ways to survive *cI-hok* counterselection. First, the *cI-hok* cassette can be eliminated by a spontaneous deletion encompassing either a part of the cassette or the entire cassette, together with the surrounding chromosomal regions at least 6.2–8.8 kb apart. The second class of mutations is the specific rearrangement within the helper plasmid producing a second copy of *cat* which is no longer CI-repressible.

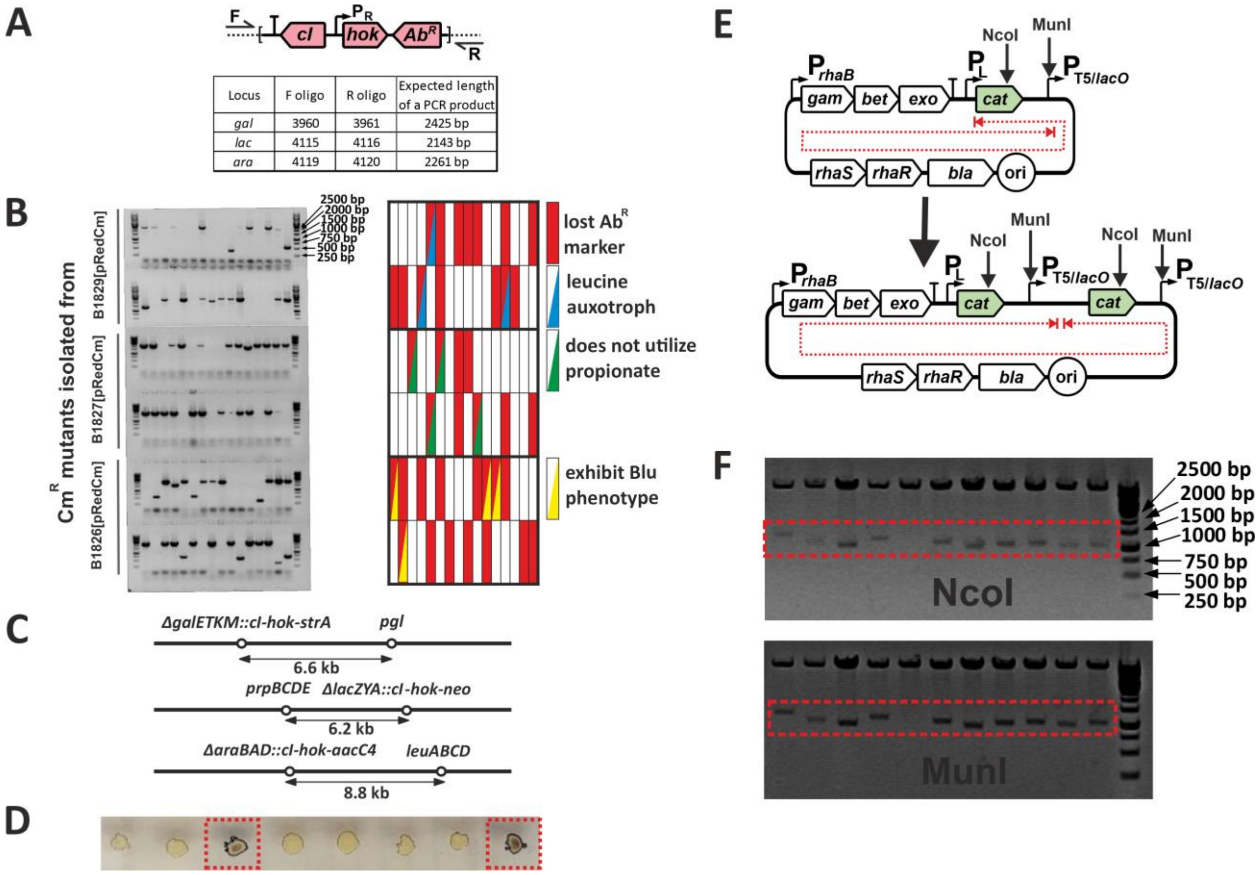

Locus	F oligo	R oligo	Expected length of a PCR product
gal	3960	3961	2425 bp
lac	4115	4116	2143 bp
ara	4119	4120	2261 bp

Supplementary Figure S6. Analysis of spontaneous mutants that survive *cI-hok* counterselection. (A) Schematic of the experiment to examine a structure of genomic loci with the inserted *cI-hok* cassette. The dashed line represents the bacterial chromosome and the solid line corresponds to the inserted *cI-hok* cassette; Ab^R – the *neo, strA*, or *aacC4* genes; F and R – forward and reverse primers. The primer pairs used for analysing each genomic locus and the expected length (for intact *cI-hok* cassette) of resulting PCR products are indicated in the table below the scheme. (B) Analysis of spontaneous Cm^R mutants by locus-specific PCR (left panel) and phenotypic examination (right panel). Briefly, 96 mutants isolated using B1826[pRedCm], B1827[pRedCm], or B1829[pRedCm] were probed by colony PCR using the primer pairs indicated in Figure S6A. The table in the left panel shows the phenotypes of the same clones in the order corresponding to that in the electrophoregram. Elimination of Ab^R marker was verified by plating mutants on LB supplemented with an appropriate antibiotic. Leucine auxotrophs failed to grow on M9-glucose medium without 50 mg/l L-leucine; *prp*-mutants were unable to grow on M9 medium plus 0.5% sodium propionate by 4 days of incubation at 37°C, but grew on M9-glucose medium. The Blu phenotype of *pgl*-mutants was examined as described previously (14). (C) Genetic linkage map of loci adjacent to the sites of *cI-hok* cassette insertion. (D) The Blu phenotype (3rd and 8th spots) conferred by the *pgl* mutation. (E) Schematic of pRedCm rearrangement, which results in *cat* duplication. The red dotted line indicates the possible scenario of rearrangement. Remarkably, 8 unique junctions (indicated by two red arrows on the bottom scheme) between *cat* and $P_{T5/lacO}$ were found among the 10 mutant plasmids. A single junction was found in three independently isolated mutants. The MunI and NcoI recognition sites within $P_{T5/lacO}$ and *cat* are unique in the case of pRedCm. These sites are duplicated in 10 of 11 mutant plasmids analysed as indicated in Figure S6F. (F) An electrophoregram of mutant plasmids digested using MunI and NcoI. The second fragment of approximately 1 kb in length (marked by the red frame) indicates duplication of the corresponding recognition sites.

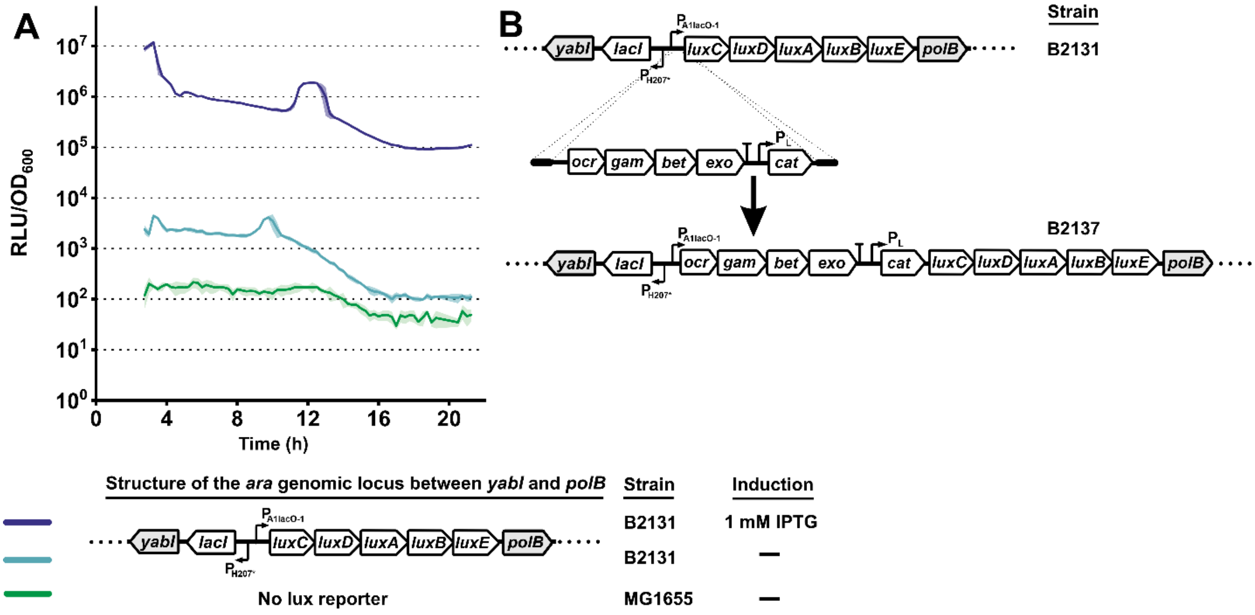

Supplementary Figure S7. Integration of the *ocr-γβexo*-P_L-*cat* helper module into the bacterial chromosome under control of the P_{H207*}-*lacI*-$P_{A1lacO-1}$ promoter-repressor cassette. (A) Verification of strong repression of the $P_{A1lacO-1}$ within the P_{H207}-*lacI*-$P_{A1lacO-1}$ cassette. The lux reporter output of the P_{H207*}-*lacI*-$P_{A1lacO-1}$ cassette fused to the *luxCDABE* operon. The asterisk in the name of the P_{H207*} promoter indicates that the native -10 site ("TATAAT") was replaced with "TATTAA" to achieve optimal LacI expression. The plotted data show a time course of luminescence measurements normalised to OD_{600}, which correspond to growth phases from the early logarithmic (OD_{600} 0.1–0.2) to late stationary phase (OD_{600} 1.7–1.9). Early time points corresponding to OD600 less than 0.1 were trimmed. Measurement was performed as described in "Measurement of *in vivo* luminescence and bacterial growth" in Supplementary Materials and Methods. The seed culture was grown overnight without induction. Upon dilution in fresh LB (at zero time point of the represented experiment) the culture was supplemented with 1 mM IPTG to induce the $P_{A1lacO-1}$ promoter as needed. The non-luminescent MG1655 strain served as an indicator for the background luminescence. The data represent the average of 6 technical replicates (6 wells inoculated from the same overnight culture), shadowed areas indicate SD. (B) Schematic illustration of chromosomal integration of the *ocr-γβexo*-P_L-*cat* module under control of the P_{H207*}-*lacI*-$P_{A1lacO-1}$ promoter-repressor pair. The resulting strain was named B2137. Grey fillings indicate native chromosomal regions, whereas white fillings depict synthetic constructs.

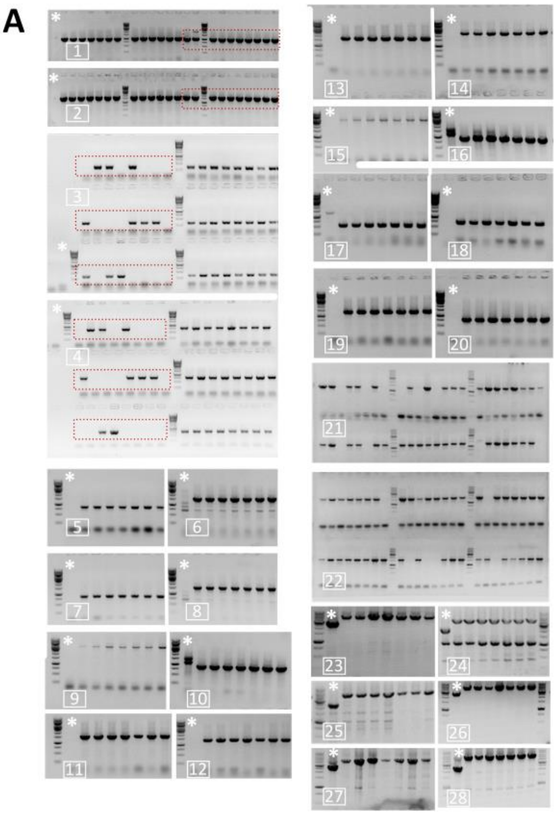

B

Integration experiment		Primers	Expected length of a PCR-product, bp	Electrophoregram
Strain	Cassette			
B1826[pRedCmOcr]	*gal::scr*	4570+3608	1039	1
		3607+4569	1643	2
B1829[pRedCmOcr]	*ara::lux*	4574+4119	332	3
		4575+4262	792	4
B1827[pRedCmOcr]	*lac::scr*	4570+4571	815	5
		862+4569	1457	6
B2141	*lac::scr*	4570+4571	815	7
		862+4569	1457	8
B2158[pRedCmOcr]	*man::scr*	2279+4569	3356	9
		4570+1653	875	10
B1829[pRedCmOcr]	*ara::scr*	4569+4262	1531	11
		4633+4570	1361	12
B2211	*pntAB-gdhA*	3435+2371	1653	13
		2229+3545	2364	14
B2163	*man::scr*	2279+4569	3356	15
		4570+1653	875	16
B2141	*lac::vio*	4573+4571	450	17
		1755+4572	644	18
B2212	*pntAB-gdhA*	4556+3435	1313	19
		4557+3545	760	20
B2212	P_{H207}-*trpE*^{S40F}*DCBA*	5301+4556	1329	21
B2211	P_{H207}-*trpE*^{S40F}*DCBA*	5301+2371	1669	22
Salmonella typhimurium LT2 *ΔgalETKM::cI-hok-neo*	*galETKM*^{wt}	5254+5255	5187 (2837 for the parental strain)	23
Salmonella typhimurium LT2 *ΔmanXYZ::cI-hok-neo*	*manXYZ*^{wt}	5260+5263	4648 (2451 for the parental strain)	24
Citrobacter freundii ATCC 8090 *ΔgalETKM::cI-hok-neo*	*galETKM*^{wt}	5278+5281	4892 (2533 for the parental strain)	25
Citrobacter freundii ATCC 8090 *ΔmanXYZ::cI-hok-neo*	*manXYZ*^{wt}	5284+5285	4923 (2736 for the parental strain)	26
Pantoea anantis SC17(0) *ΔgalTKM::cI-hok-neo*	*galTKM*^{wt}	5266+5267	4174 (2833 for the parental strain)	27
Pantoea anantis SC17(0) *ΔmanXYZ::cI-hok-neo*	*manXYZ*^{wt}	5272+5275	4780 (2521 for the parental strain)	28

C

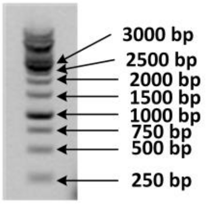

Supplementary Figure S8. Verification of the correct integration of linear cassettes into targeted genomic loci by locus-specific PCR. (A) PCR products probing the junctions between genomic and cassette sequences resolved by gel electrophoresis. Two electropherograms, except for the experiments with *Salmonella typhimurium* LT2, *Citrobacter freundii* ATCC 8090, and *Pantoea ananatis* SC(0), correspond to a single integration experiment outlined in Figure S8B. In the case of these bacteria, the presence of the desired modification was tested using a single pair of external primers. The number in the white square is the index number of the electropherogram. A red dotted frame indicates clones that were recognised as non-recombinant upon phenotypic screening but successfully acquired a cassette in the correct chromosomal site. The remaining clones were randomly picked among those that exhibited a recombinant phenotype conferred by a cassette to be inserted. White asterisks indicate a negative control PCR, conducted using a colony of parental strains subjected to cassette integration. (B) The table outlines the PCRs used to verify the correct integration of a cassette in a targeted genomic locus. The primer pairs for amplification of junctions between the genomic sequence and cassette sequence as well as the expected lengths of resulting PCR-products are listed. The index numbers of electropherograms correspond to those indicated in Figure S8A. (C) GeneRuler 1 kb DNA Ladder (Thermo Fisher Scientific) used as a marker for gel electrophoresis.

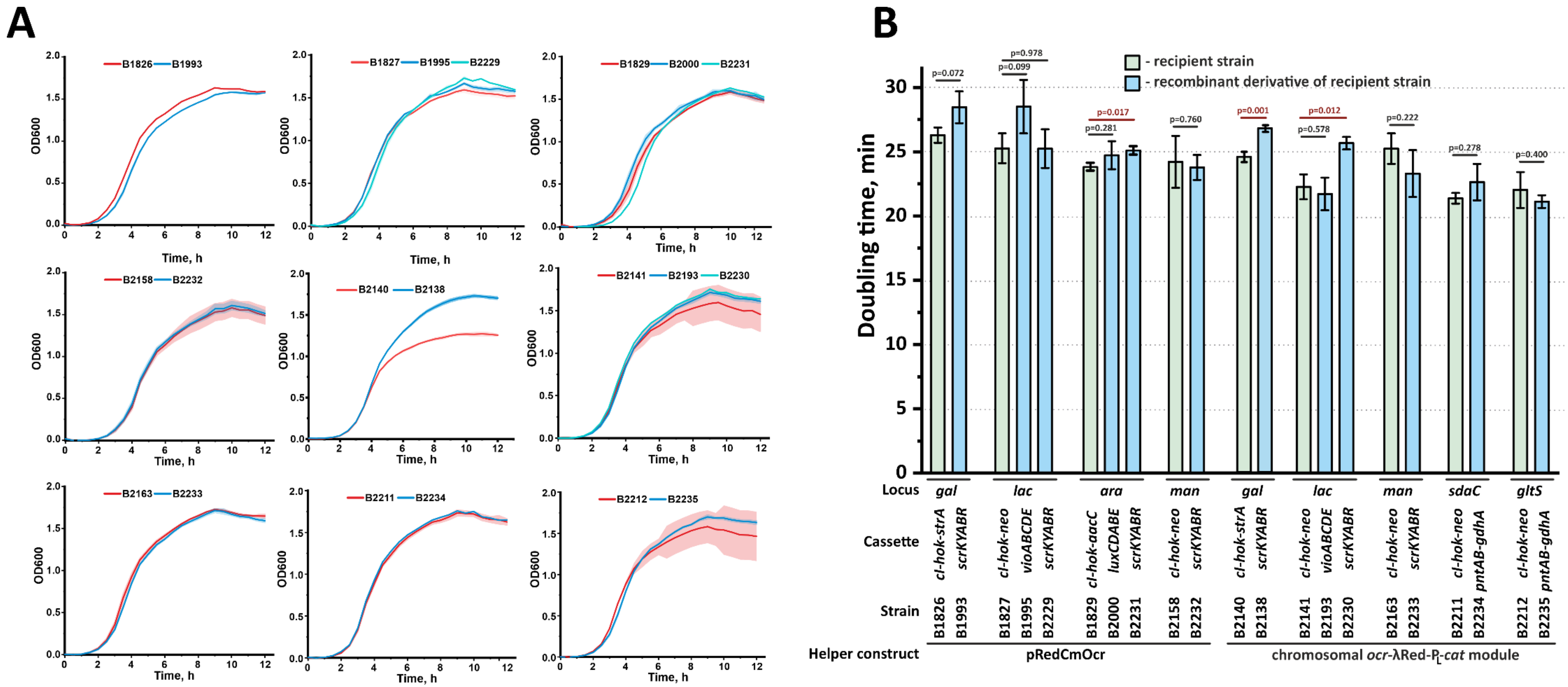

Supplementary Figure S9. Comparison of the growth of recipient strains used for integration of the *scr, vio, lux,* and *pntAB-gdhA* cassettes with the resulting recombinant strains. (A) Growth curves of the tested strains in LB medium without supplements. The information on the genotypes of these strains, i.e., the mutated loci and cassette inserted, is indicated in the legend under the horizontal axis in panel (B). Complete genotypes are listed in Supplementary Table S2. The experiment was performed as described in the section “Measurement of *in vivo* luminescence and bacterial growth” in the Supplementary Material and Methods, except the bioluminescence was not monitored. For experiments with pairs that include strains constructed using the pRedCmOcr helper, both a recipient strain and its recombinant derivative were transformed with this plasmid and the seed cultures were grown in presence of 200 mg/l ampicillin. The shown growth curves are the averages of three technical replicates (three wells inoculated from a single overnight culture), shadowed areas indicate the SD. (B) Doubling times were calculated using the growth curves shown in (A). The values shown are the averages of three technical replicates; error bars indicate SD. The values were compared using t-test with unequal variances. Two-sided p-values are shown. The p-values highlighted in red indicate pairs where the difference in the average doubling times was significant at a significance level of 0.05.

3. *cI-hok* counterselection facilitates the transfer of markerless genomic loci

The only exception from the generally good results was an attempt to replace the *ΔlacZYA::cI-hok-neo* cassette with the *lacZYA* operon in the B2141 strain, which yielded only 6–13% of lactose-utilizing recombinants. However, the same locus could be readily transferred to the isogenic B1827 strain. These observations lead to the assumption that some interaction occurs between the specific type of incoming DNA and helper construct. The chromosomal *ocr-γβexo*-P_L-*cat* helper module in the chromosome of B2141 is placed under the control of $P_{A1lacO-1}$ promoter and the second copy of the *lacI* gene, which is overexpressed because the native promoter is replaced with the strong P_{H207} promoter. Remarkably, the expression level of LacI is so high that strains harbouring this chromosomal construct and the intact *lacZYA* operon exhibit a partial Lac^- phenotype on tetrazolium-lactose agar (data not shown). We hypothesized, that the drop in the percentage of positive recombinants in the case of transferring the *lacZYA* operon to the B2141 strain can be caused by some interaction between LacI and its binding sites within the incoming DNA. Indeed, when trying to transfer the *Δ[lacI-lacZYA]::scrKYABR* mutation from B2229 into B2141 we observed that the percentage of positive recombinants was close to 100%. Notably, the *Δ[lacI-lacZYA]::scrKYABR* construct has all three *lac*-operators deleted.

A possible explanation for this observation is a metabolic burden imposed by expression of the *lacZYA* operon, which retards the growth Lac^+ recombinants during outgrowth in the liquid media, thereby reducing their apparent percentage among total chloramphenicol-resistant survivors. However, this is unlikely as *lacZYA* is tightly repressed in the derivative of the B2141 strain owing to the elevated concentration of LacI. We also examined this assumption experimentally. We compared the growth rates of the B2141 strain and isogenic B2137 strain, which possess the intact *lacZYA* operon (Supplementary Figure S10). Our results again indicate the metabolic burden to be an unlikely explanation.

The interaction between LacI and its binding sites is further supported by the several relatively unsuccessful attempts (yielding approximately 20% of the positive recombinants) to introduce the *vioABCDE* cassette in *lac* loci using the B2141 strain (Figure 4C in the main manuscript). Simultaneously, this cassette recombined normally in experiments with the isogenic B1827 strain and the pRedCmOcr helper. Remarkably, one of the homology arms of this cassette is within the *lac*-promoter and comprises the *lacO* sequence. Indeed, tight binding of LacI to the homology arm can hinder DNA processing by Red proteins as well as further recombination.

A strong counterargument against the role of LacI is that it cannot affect recombination distant from its binding site, as in the case of P1 transduction or genomic DNA electroporation when the incoming fragment is 90 kb and 30 kb in length, respectively. Therefore, further work is required to resolve this question. We can only conclude that the specific combination of the preexisting chromosomal construct and incoming DNA containing the wild-type *lac* operon is the cause of the observed problem. In practice, this could be easily circumvented by a rational choice of the proper helper.

We also found that, compared with P1 transduction, electroporation of the recipient strains with the donor genomic DNA yielded 10–30 times more Cm^R colonies (see data represented in Supplementary Tables S6 and S7) and generally, a slightly higher percentage of positive recombinants. Finally, induction of λRed stimulated allelic exchange in electroporation experiments, whereby the total number of recombinants increased 5–10 times compared to that obtained with uninduced cells (data not shown).

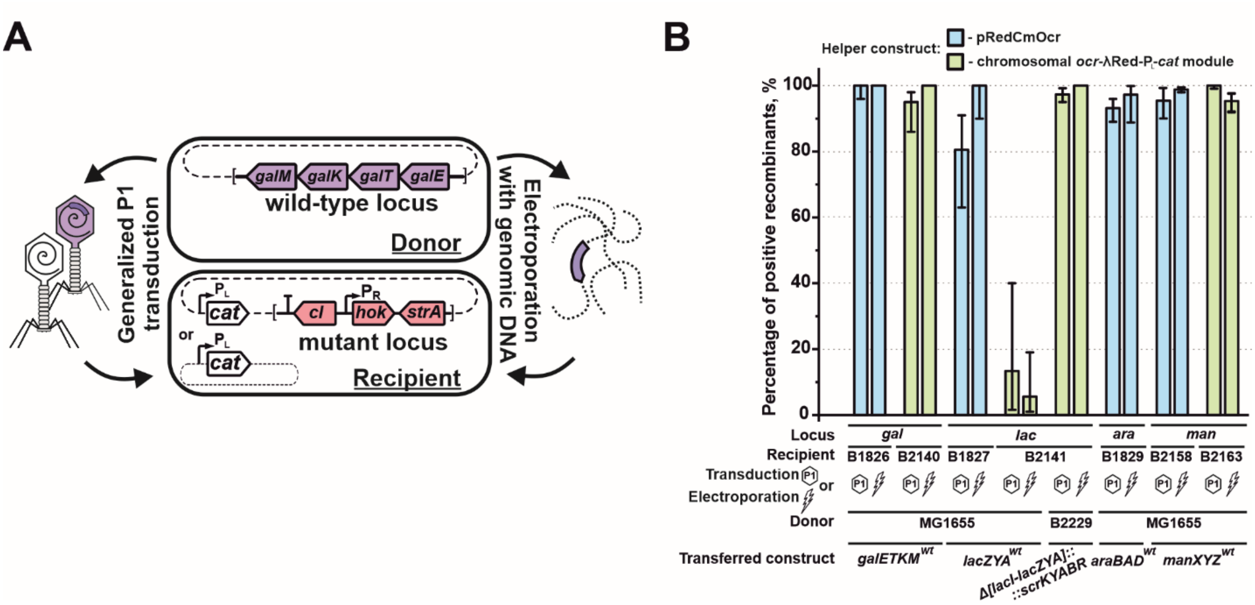

Supplementary Figure S10. *cI-hok* counterselection enables the facile transfer of unmarked genomic loci. (A) Schematic of experiments for the markerless transfer of chromosomal regions (the *galETKM* operon, in this case) between strains. These regions from the chromosome of a donor strain are delivered by either P1 transduction or direct electroporation, to a recipient cell having the *cI-hok* cassette inserted in a cognate region. Upon recombination, the *cI-hok* cassette is replaced with the transferred region, whereby recombinant colonies can be selected for Cm^R due to derepression of the P_L-*cat* module within pRedCmOcr or the chromosomal *ocr-γβexo*-P_L-*cat* helper construct. (B) The efficiency of markerless transfer across the *gal, lac, ara,* and *man* genomic loci. The recipient strain was infected with 0.5×10^8–1.0×10^8 pfu of P1*vir* grown using MG1655 or B2229 donor strains.

Alternatively, 2–2.5 µg of genomic DNA (sheared to approx. 30 kb via silica column purification) from these strains was electroporated into recipient cells induced for expressing the *ocr-γβexo* operon, followed by overnight recovery and recombinant selection on chloramphenicol-supplemented tetrazolium agar with D-galactose, lactose, L-arabinose, or sucrose (for more details see "Markerless transfer of genomic loci by P1 transduction or transformation with genomic DNA" in the Supplementary Materials and Methods). Cm^R colonies regaining the ability to utilise these carbohydrates were recognised as recombinants. The percentage of positive recombinants was calculated by dividing the titre of recombinants by the total number of Cm^R colonies. The exact numerator and denominator are listed in Supplementary Tables S6 and S7. The values represent the result of a single biological replicate, whereas error bars indicate 95% Clopper-Pearson confidence intervals for a proportion.

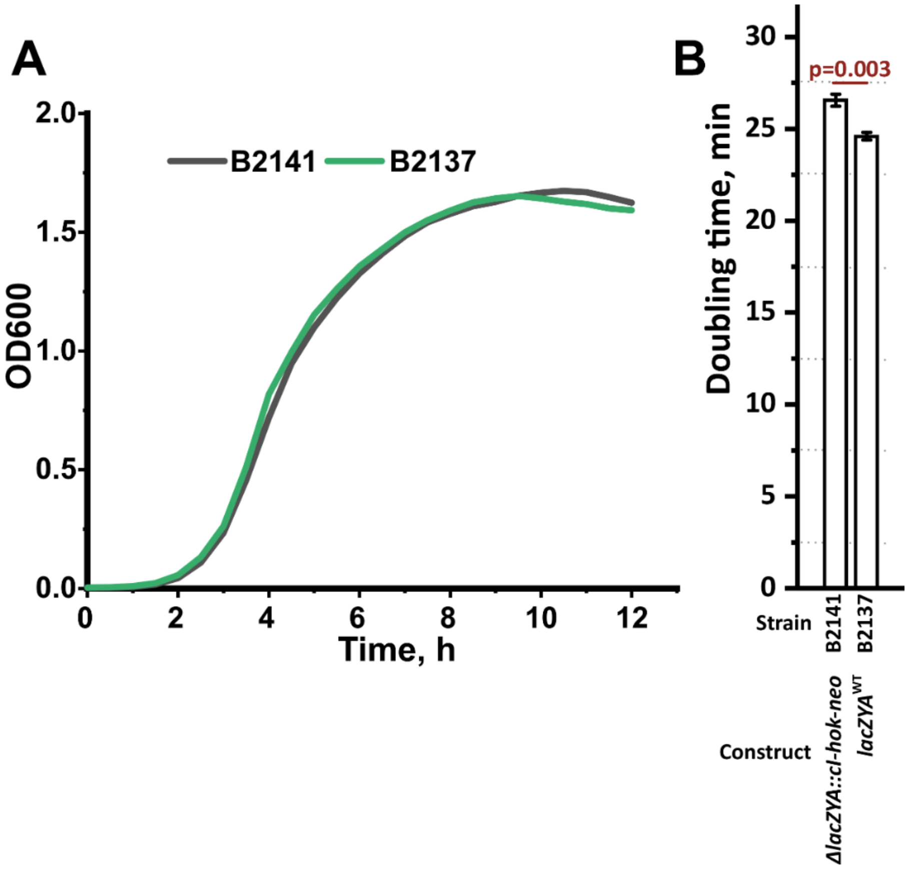

Supplementary Figure S11. Comparison of the growth of the B2141 and B2137 strains. (A) Growth curves of the tested strains in LB medium without supplements. The experiment was performed as described in the section "Measurement of *in vivo* luminescence and bacterial growth" in the Supplementary Material and Methods, except the bioluminescence was not monitored. The shown growth curves are the averages of three technical replicates (three wells inoculated from a single overnight culture); SD is shown but is small and does not extend beyond the solid line. (B) Doubling times for the B2141 and B2137 strains were calculated using the growth curves shown in (A). The values shown are the averages of three technical replicates; error bars indicate SD. The values were compared using t-test with unequal variances. A two-sided p-value is shown.

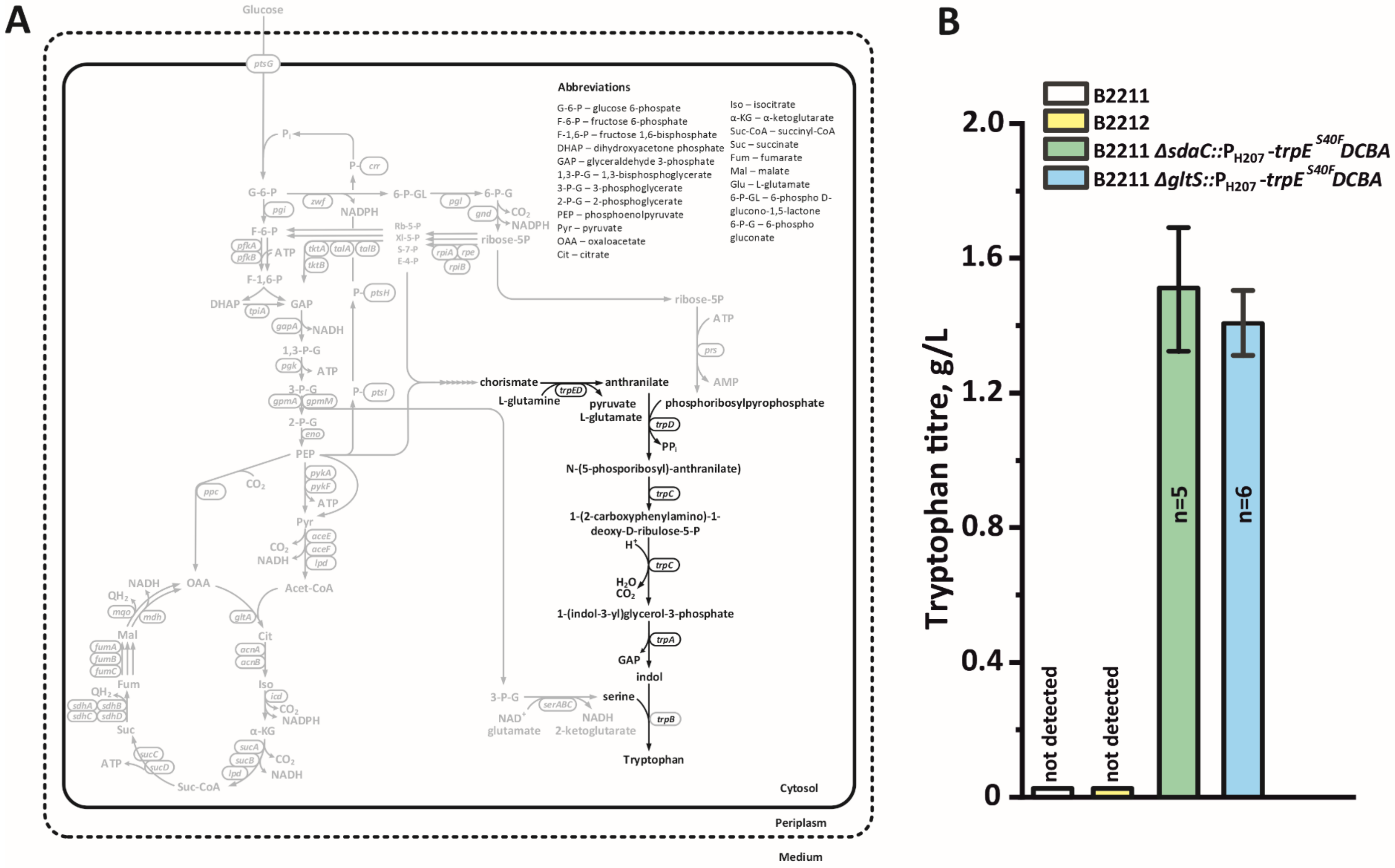

Supplementary Figure S12. Metabolic engineering of an L-tryptophan producing strain. (A) A metabolic map of the L-tryptophan biosynthesis pathway. Black lines and text indicate the reactions and enzymes affected by insertion of the P_{H207}-*trpE*^{S40F}*DCBA* construct in the chromosome of the B2211 and B2212 strains. (B) L-tryptophan accumulation upon the cultivation of the B2211 *ΔsdaC::*P_{H207}-*trpE*^{S40F}*DCBA* and B2212 *ΔgltS::*P_{H207}-*trpE*^{S40F}*DCBA* strains. Strain cultivation and L-tryptophan quantification were performed as described in the section "Estimating the capacity of strains for amino acids accumulation" in the Supplementary Materials and Methods. The values shown are the averages of the indicated number of biological replicates; error bars indicate SD.

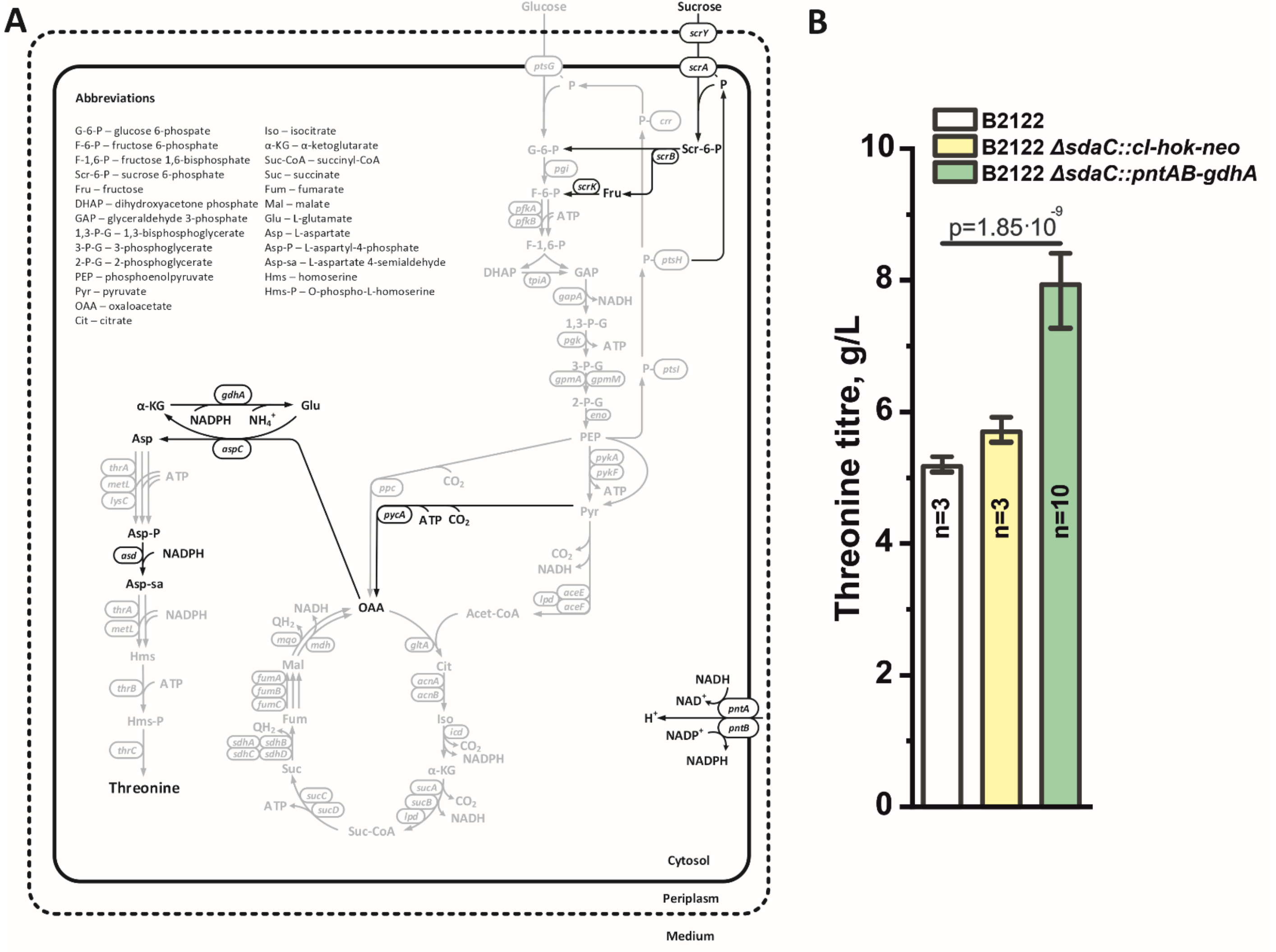

Supplementary Figure S13. Metabolic engineering of an L-threonine producing strain. (A) A metabolic map of the L-threonine biosynthesis pathway. Black lines and text indicate the reactions and enzymes affected by insertion of the *pntAB-gdhA* construct in the chromosome of the B2122 strain. (B) L-threonine accumulation upon cultivation of the B2211 *ΔsdaC::pntAB-gdhA* and parental strains. Strain cultivation and L-threonine quantification were performed as described in the section “Estimating the capacity of strains for amino acids accumulation” in the Supplementary Materials and Methods. The values shown are the averages of the indicated number of biological replicates; error bars indicate SD. The values were compared using t-test with unequal variances. A two-sided p-value is shown.

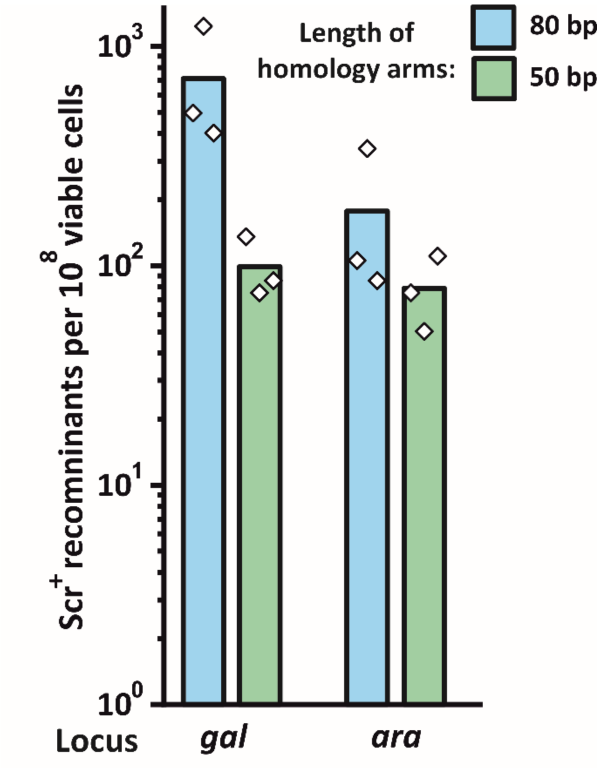

Supplementary Figure S14. Efficiency of replacing the *galETKM* and *araBAD* operons with the *scrKYABR* cassettes containing either 80-bp or 50-bp homology arms. MG1655 cells carrying pRedCmOcr were induced for expression of λRed and Ocr functions and electroporated with 0.1 µg of the *scrKYABR* cassette. Scr^+ recombinants were selected on M9 plus 0.5% sucrose plates and the viable cell titre was determined on non-selective LB plates. The sequences of the cassettes are listed in Supplementary Table S4 under the names "*gal::scrKYABR*^{80}", "*gal::scrKYABR*^{50}", *ara::scrKYABR*^{80}", "*ara::scrKYABR*^{50}". The values shown are the average of three technical replicates; open diamonds indicate the exact values for each replicate. The same batch of competent cells was used for all electroporation experiments.

SUPPLEMENTARY REFERENCES

1. Bubnov, D.M., Yuzbashev, T.V., Vybornaya, T.V., Netrusov, A.I. and Sineoky, S.P. (2018) Development of new versatile plasmid-based systems for lambdaRed-mediated Escherichia coli genome engineering. *J. Microbiol. Methods*, **151**, 48–56.

2. Vybornaya, T.V., Yuzbashev, T.V., Fedorov, A.S., Bubnov, D.M., Filippova, S.S., Bondarenko, F.V. and Sineoky, S.P. (2020) Use of an Alternative Pathway for Isoleucine Synthesis in Threonine-Producing Strains of Escherichia coli. *Appl. Biochem. Microbiol.*, **56**, 759–769.

3. Sergueev, K., Yu, D., Austin, S. and Court, D. (2001) Cell toxicity caused by products of the p(L) operon of bacteriophage lambda. *Gene*, **272**, 227–35.

4. Murphy, K.C. (1991) Lambda Gam protein inhibits the helicase and chi-stimulated recombination activities of Escherichia coli RecBCD enzyme. *J. Bacteriol.*, **173**, 5808–5821.

5. Datta, S., Costantino, N. and Court, D.L. (2006) A set of recombineering plasmids for gram-negative bacteria. *Gene*, **379**, 109–15.

6. Sarkar, S., Ma, W.T. and Sandri, G.H. (1992) On fluctuation analysis: a new, simple and efficient method for computing the expected number of mutants. *Genetica*, **85**, 173–9.

7. Foster, P.L. (2006) Methods for determining spontaneous mutation rates. *Methods Enzym.*, **409**, 195–213.

8. Radchenko, E.A., McGinty, R.J., Aksenova, A.Y., Neil, A.J. and Mirkin, S.M. (2018) Quantitative Analysis of the Rates for Repeat-Mediated Genome Instability in a Yeast Experimental System. In Muzi-Falconi,M., Brown,G.W. (eds), *Genome Instability: Methods and Protocols*, Methods in Molecular Biology. Springer, New York, NY, pp. 421–438.

9. Luria, S.E. and Delbrück, M. (1943) Mutations of Bacteria from Virus Sensitivity to Virus Resistance. *Genetics*, **28**, 491–511.

10. Yu, D., Sawitzke, J.A., Ellis, H. and Court, D.L. (2003) Recombineering with overlapping single-stranded DNA oligonucleotides: testing a recombination intermediate. *Proc Natl Acad Sci U A*, **100**, 7207–12.

11. Thomason, L.C., Costantino, N. and Court, D.L. (2007) E. coli Genome Manipulation by P1 Transduction. *Curr. Protoc. Mol. Biol.*, **79**, 1.17.1-1.17.8.

12. Lanzer, M. and Bujard, H. (1988) Promoters largely determine the efficiency of repressor action. *Proc Natl Acad Sci U A*, **85**, 8973–7.

13. Lutz, R. and Bujard, H. (1997) Independent and tight regulation of transcriptional units in Escherichia coli via the LacR/O, the TetR/O and AraC/I1-I2 regulatory elements. *Nucleic Acids Res.*, **25**, 1203–1210.

14. Thomason, L.C., Court, D.L., Datta, A.R., Khanna, R. and Rosner, J.L. (2004) Identification of the Escherichia coli K-12 ybhE gene as pgl, encoding 6-phosphogluconolactonase. *J. Bacteriol.*, **186**, 8248–8253.
